# Extracellular matrix in skeletal muscle injury and atrophy: mechanisms and therapeutic implications

**DOI:** 10.1016/j.jot.2025.03.004

**Published:** 2025-05-16

**Authors:** Xiaoyang Ge, Yesheng Jin, Jingyuan He, Zhihao Jia, Ying Liu, Yong Xu

**Affiliations:** a Department of Orthopaedics, The First Affiliated Hospital of Soochow University, Orthopedic Institute, MOE Key Laboratory of Geriatric Diseases and Immunology, Suzhou Medical College, Soochow University, Suzhou, Jiangsu, 215000 China; b Cambridge-Suda Genomic Resource Center, Suzhou Medical College, Soochow University, Suzhou, 215000, China; c Wuxi School of Medicine, Jiangnan University, Wuxi, Jiangsu, China

**Keywords:** Extracellular matrix, Skeletal muscle, Muscle injury, Muscle atrophy, Tissue engineering, Muscle repair

## Abstract

Extracellular matrix (ECM) is an intricate, dynamic network that is essential for structural and biochemical support of skeletal muscle cells. Upon skeletal muscle injury, ECM undergoes rapid remodeling to clear damaged tissue and provides a scaffold to support muscle regeneration. Disruptions in the structure and composition of ECM lead to fibrosis and impaired muscle function, consequently hindering the regenerative capability of skeletal muscle following acute injury. Besides, dysregulation of ECM can also affect muscle mass and cross-sectional area, contributing to the onset of muscle atrophy. Thus, understanding the physiological and mechanical roles of ECM in skeletal muscle injury and atrophy is crucial for developing strategies to treat muscle-related diseases. This review focuses on the complex interactions between the ECM and skeletal muscle, aiming to summarize the regulatory function and mechanism of ECM in muscle development, injury repair, and atrophy. Additionally, it covers recent advances in the treatment of skeletal muscle diseases via the utilization or modulation of ECM components. We will discuss the potential benefits of ECM-based therapies and the current challenges in this area, including producing standardized ECM, minimizing graft-versus-host disease (GVHD), and ensuring that scaffolds have the appropriate biological function. In sum, this comprehensive review will provide a foundation and insights into the relationship between ECM and skeletal muscle, shedding light on the development of ECM-based therapies in the treatment of muscle injury and atrophy.

**The Translational Potential of This Article:**

This article systematically explores the regulatory function and mechanism of ECM in muscle development, injury repair, and atrophy. It also summarizes recent advances in therapeutic strategies for skeletal muscle injury and atrophy from the ECM perspective. Insights from this review contribute to the development of therapeutic strategies for skeletal muscle injury and atrophy by modulating or utilizing ECM components, thus providing novel therapeutic avenues for tissue engineering and regenerative medicine approaches to muscle-related disorders.

## Graphical abstract

**Figure:**
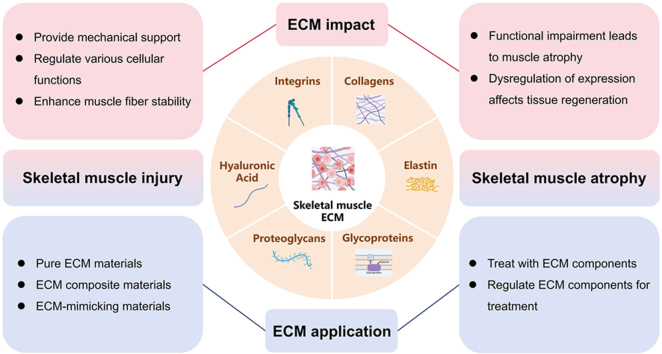

## 1 Introduction

The extracellular matrix (ECM) is an essential component of tissue structure and function, providing not only mechanical strength but also transmitting biochemical signals to cells [1]. It comprises the three-dimensional (3D) architecture of muscle fibers and modulates cell adhesion, migration, and differentiation through its interactions with cell surface receptors [2]. The skeletal muscle tissue ECM contains proteins, polysaccharides, and proteoglycan groups.

Skeletal muscle injury is a common musculoskeletal disorder. However, muscle repair is a complex, multistep process that requires a cascade of biochemical reactions and cellular events. In response to muscle fiber injury, the ECM's constituents and architecture undergo rapid transformation. These transformations include the degradation of existing ECM components and the synthesis of new ECM, which are necessary to provide spatial and architectural support for muscle repair. However, incorrect structural remodeling of the ECM has been demonstrated to be associated with muscle fibrosis [3]. Irregularities of the ECM are also linked to the development of skeletal muscle atrophy. Skeletal muscle atrophy, characterized by muscle wasting and performance impairment, occurs due to inactivity, diseases, or aging. In the aging or disease state, the ECM is affected by increased stiffness and collagen synthesis, ultimately having a negative impact on the capacity for myogenic differentiation of muscle satellite cells (MuSCs). Consequently, ECM modulation and remodeling may potentially ameliorate skeletal muscle atrophy [4].

In this review, we will detail the biological properties of the ECM and discuss its impact on skeletal muscle injury and atrophy. We will then explore the applications of ECM utilization and regulation in the treatment of these conditions.

## 2 Skeletal muscle and its related diseases

### 2.1 Composition, structure, and classification of skeletal muscle

Skeletal muscle, the efficient engine of motor function, constitutes 40 %–50 % of the human body mass. All movements performed by the human body require the coordinated action of a series of muscle groups, and the health of skeletal muscles significantly influences the healthspan [5]. Skeletal muscles are characterized by their unique structure, which manifests as the orderly alignment of muscle fibers [6]. Muscle fibers, also known as myocytes, are formed by the fusion of myoblasts, with their nuclei located peripherally, adjacent to the plasma membrane. Inside the muscle fibers are numerous myofibrils, which contain a vast array of myofilaments. These myofilaments are arranged in an orderly manner to form sarcomeres, the basic units governing muscle contraction. Muscle fibers are primarily composed of contractile proteins, regulatory proteins, cytoskeletal proteins, and myoplasm. Muscle contraction primarily relies on the interaction of contractile proteins, including actin and myosin [7].

In mammalian skeletal muscle, two major types of muscle fibers are present: Type I and Type II, while Type II can be further classified into Type IIA, Type IIX, and Type IIB fibers. Type I fibers, known as slow-oxidative fibers, are characterized by the presence of numerous mitochondria and high antioxidative capacity, which makes them more resistant to fatigue. Consequently, they are well suited for long, low–intensity activities with longer contraction durations but slower speeds. In contrast, Type II focuses on anaerobic glycolysis as their main source of energy production, which allows these muscle fibers to perform quickly and efficiently; however, they are more prone to fatigue [8]. The type and proportion of muscle fibers can change in response to functional demands. Exercise training is one of the main factors affecting muscle fiber type, mediating muscle growth and adaptation by activating intracellular signaling pathways [9]. Given the distinct differences in exercise adaptability, metabolic characteristics, and resistance to fatigue between Type I and Type II muscle fibers, future research on skeletal muscle regeneration should focus on identifying the types of regenerated muscle fibers to better understand the functional recovery. In addition, unraveling the mechanism for the regeneration of different types of fibers is also required. Thus, a potential future avenue is to modulate the type of regenerated muscle fibers through distinct strategies, such as the regulation of exercise stimulus frequency or the regenerative environment. Thereby, to restore both the physical and physiological properties of the regenerated skeletal muscle in the particular region.

### 2.2 Muscle injury and regeneration

Skeletal muscle possesses significant regenerative ability and is commonly used as a model to study adult muscle regeneration. Skeletal muscle injury is typically induced by direct mechanical stress during muscle contraction, such as intense exercise, overuse, impact, laceration, or exposure to toxins. The relatively superficial location of muscles makes them more susceptible to these acute injuries. Damage can also be caused by impairment of the muscle cell membrane, resulting in dysregulated calcium ion influx, activation of proteases and hydrolases, and further exacerbation of muscle injury [10].

**Fig. 1:**
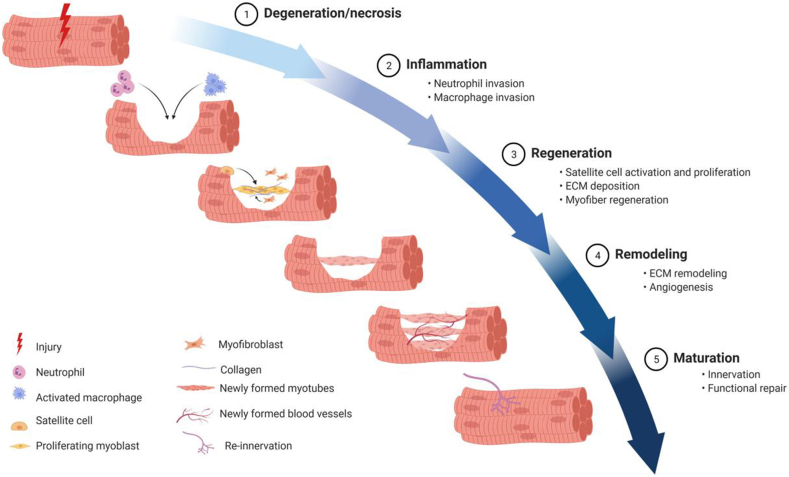
Skeletal muscle possesses an innate regenerative capacity, with its restorative processes interlinked and temporally orchestrated. The process begins with muscle necrosis, which triggers an inflammatory response, paving the way for regeneration. Subsequently, the tissue undergoes a phase of remodeling, ultimately reaching a stage of maturation that results in the full restoration of muscle function [12].

The regeneration of skeletal muscle involves MuSCs activation and proliferation, inflammatory response, and myofiber formation. The regenerative process of skeletal muscle typically goes through three phases. First, the inflammatory phase, characterized by myofiber necrosis and an accompanying inflammatory cell response; second, the repair phase, during which new myofibers and scar tissue begin to form; and finally, the remodeling phase, which involves the reconstruction of muscle vasculature and nerve connections (Fig. 1) [11].

Post-injury, inflammatory cells rapidly enter the damaged area to clear cellular debris and activate MuSCs. Early-recruited pro-inflammatory M1 macrophages facilitate the clearance of debris from the damaged area and express Th1 cytokines. These M1 macrophages peak 1–2 days post-injury and then transition to anti-inflammatory M2 macrophages. This change in macrophage type signifies the transition of skeletal muscle from the inflammatory to the repair phase. To advance to the reparative phase, M2 macrophages secrete interleukin-10 (IL-10) and transforming growth factor-beta (TGF-β) to limit over-excited pro-inflammatory responses. On the other hand, insulin-like growth factor-1 (IGF-1) and fibroblast growth factors (FGF) are synthesized and stimulate the differentiation of MuSCs [13].

The preservation of skeletal muscle structure is highly dependent on the activities of MuSCs [14]. Subsequent to injury, following the activation with cytokines and growth factors, MuSCs start proliferating and eventually mature into myocytes, which form the central-nucleated newly regenerated myofibers. The activated MuSCs express MyoD and Myf5 to regulate their further activation and proliferation [15].

After a successful repair phase, the regenerative process of skeletal muscle shifts to the remodeling phase. This phase primarily involves the remodeling of the ECM. Myofibroblasts are cells that produce the ECM, deposit collagen during the inflammatory phase to provide a scaffold for the newly formed regenerating myofibers, supporting the transition to the regeneration phase [16]. In addition, the number of vascular endothelial cells gradually increases during this phase, releasing various growth factors, including angiopoietin-1 [17], IGF-1 [18], hepatocyte growth factor (HGF) [19], and vascular endothelial growth factor (VEGF) [20] to regulate the differentiation of MuSCs. In the terminal differentiation phase of myofibers, motor nerve fibers regenerate, furthering the recovery of muscle strength and coordination [21].

### 2.3 Muscle atrophy and treatments

The cytoplasm of skeletal muscle cells is densely packed with contractile proteins, mitochondria and endoplasmic reticulum. This dense arrangement leaves no spare space, meaning that protein and organelle turnover substantially influences muscle fiber dimensions and functionality. Under the stimulation of exercise or anabolic hormones, muscles grow by accumulating novel proteins and organelles within the cytoplasm, a process known as muscle hypertrophy or overcompensation. Conversely, catabolic conditions lead to a reduction in the aforementioned cellular contents, resulting in a decrease in cell volume, a condition referred to as atrophy [22].

Muscle atrophy in skeletal muscle can be classified into primary and secondary types. Primary skeletal muscle atrophy is typically associated with inflammation, metabolic dysfunction of muscle fibers, muscle spasms, or stiffness. Physiological responses such as fasting or malnutrition can also induce muscle atrophy. Secondary skeletal muscle atrophy arises as a consequence of systemic afflictions, such as sarcopenia related to aging, cachexia caused by cancer [23].

**Fig. 2:**
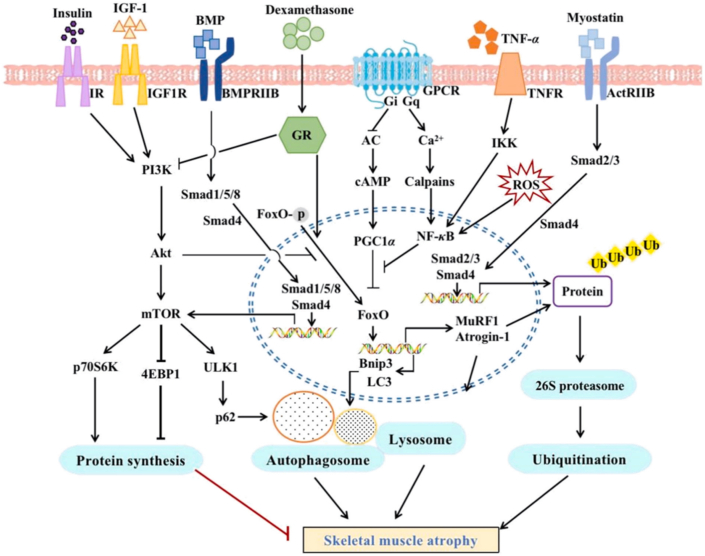
Skeletal muscle atrophy is a multifactorial process that essentially results from a disturbance in the equilibrium between protein anabolism and catabolism. The disturbances include oxidative stress, overstimulation of both the ubiquitin-proteasome and autophagy-lysosome systems, overactivation of calpain and caspase proteinases, and the inhibition of the mTOR signaling pathway. The combined activity of these regulatory systems leads to muscle fiber atrophy, which consequently results in less muscle power and endurance [30].

Skeletal muscle atrophy involves reduction in muscle bulk and diameter of muscle fibers, culminating in a decline in muscle contractile force, an increase in the propensity for fatigue, and a diminished capacity for physical activity [24]. Numerous signaling cascades are instrumental in the modulation of muscle wasting, such as the insulin/IGF-1-Protein Kinase B (AKT)-mammalian Target of Rapamycin (mTOR) axis, which facilitates protein anabolism and suppresses proteolysis [25]. The TGFβ/Myostatin/Activin/Bone Morphogenetic Proteins (BMP) pathway controls muscle mass by regulating Sma and Mad related proteins (SMAD) 2/3 and SMAD 1/5/8 transcription factors [26]. β-adrenergic signaling modulates muscle mass through the AKT-mTOR axis [27]. Metabolic regulatory factors like AMP-activated protein kinase (AMPK) [28] and Peroxisome Proliferator-Activated Receptor Gamma Coactivator 1-alpha (PGC1α) [29] are integral to muscle growth (Fig. 2).

Therapeutic strategies for skeletal muscle atrophy include exercise therapy, nutritional supplementation, pharmacological interventions, gene and stem cell-based treatments, and modulation of myokines [30]. Among these strategies, exercise is considered the most efficacious and feasible method, encompassing resistance training and endurance training. Endurance training, particularly aerobic exercise training (AET), activates the AKT/mTOR signaling cascade, promoting protein synthesis and inhibiting muscle atrophy. AET also reduces the secretion of pro-inflammatory cytokines and stimulates the activity of glutathione peroxidase 1 and catalase, thereby decreasing reactive oxygen species -induced stress and inflammatory responses, and mitigating the degradation of muscle proteins [31]. Current therapeutic approaches for skeletal muscle atrophy are limited by applicability, individual variability, patient compliance, and safety. To date, there is still a lack of broadly efficacious curative methods.

## 3 The crosstalk between ECM and skeletal muscle

### 3.1 Overview of ECM

The ECM, an essential acellular scaffold that pervades all tissues, serves as a critical foundation for a plethora of biological processes. It facilitates cell adhesion, migration, differentiation, and the intricate organization of tissues. Composed predominantly of collagens, elastins, fibronectins, laminins, proteoglycans, and glycoproteins, the ECM establishes a three-dimensional framework that underpins cellular interactions [32]. The ECM of skeletal muscle is primarily constituted by collagen I and collagen III fibers. Collagen I forms the primary fibrous network of the muscle, providing mechanical strength and stability. Collagen III forms a loosely arranged fibrous network, providing elasticity and compliance [33]. Through interactions with integrins, the ECM also exerts a significant influence on the regulation of cellular physiological behaviors [34]. The intact ECM structure is vital to the efficient propagation of contractile forces along and across muscle fibers, thereby enhancing muscle contraction efficiency and safeguarding muscle fibers against detrimental mechanical stress. In summary, the ECM exerts a pivotal regulatory influence over the entire developmental process and functionality of skeletal muscle.

### 3.2 ECM of injured skeletal muscle

After skeletal muscle injury, the ECM undergoes rapid changes. At the onset of injury, the disruption of the muscle cell membrane triggers an inflammatory response. Neutrophils and macrophages are recruited to the lesioned tissue, subsequently secreting a cadre of cytokine and MMPs. MMPs degrade collagen in the ECM, thereby creating space for muscle repair [35]. The tissue destruction resulting from inflammatory responses also activates and facilitates the expansion of MuSCs. Activated MuSCs enhance the synthesis of collagen I and collagen III, which facilitate the recuperation of muscular tissue integrity [36]. The basement membrane, predominantly composed of collagen IV, is also reconstructed, providing attachment sites for muscle cells and facilitating cell migration and differentiation, thereby offering structural support necessary for the generation of new muscle fibers [37]. Incomplete or inappropriate process of ECM leads to muscle fibrosis, characterized by excessive deposition and abnormal cross-linking of collagen [38].

### 3.3 ECM in atrophic skeletal muscle

Skeletal muscle atrophy exhibits features of modifications in the structure, biochemistry, cellularity, and functionality of the ECM, often accompanied by a deterioration of various muscle performance parameters [39]. In the aging skeletal muscle, with pathways associated with ECM remodeling being broadly downregulated. This indicates that the age-associated sarcopenia is typically coupled with a decline in the formation and preservation of the ECM [40]. Specifically, as age advances, both the content and the degree of collagen I crosslinking increase, resulting in enhanced muscle stiffness. The increased stiffness of the ECM affects integrins and mechanoreceptors on the cell surface, activating the TGF-β/SMAD cascade, which results in muscle fibrosis and a decline in function. Moreover, the increased rigidity of the ECM also impacts the functionality of MuSCs, thereby resulting in an impaired potential for muscular regeneration. Alterations in the ECM physical properties also impair mitochondrial function via mechanotransductive forces, culminating in heightened reactive oxygen species generation and a concomitant reduction in antioxidant defenses, thereby aggravating ECM dysregulation [4].

## 4 The impact of ECM on skeletal muscle

Within the complex control of skeletal muscle, the ECM is a critical element, directing the structural integrity and the functional capacity of muscle tissue. This part explores the multifarious functions of ECM components, such as collagen, proteoglycans, integrins and hyaluronic acid (HA), in shaping the foundational architecture of skeletal muscle and sustaining the physiological functions, thereby laying the theoretical foundation for ECM therapeutic utilization in skeletal muscle disorders.

Collagen, as the key structural protein within the ECM, is indispensable for the preservation of the muscle tissue framework and muscular activities. It not only provides structural support and stability to the muscle but also actively participates in key biological processes [4]. The regulation of collagen quantity and alignment during ECM remodeling serves to diminish parallel stiffness and augment serial stiffness, thereby enhancing the contractile force and strength of engineered skeletal muscle. Prior work has illustrated that alternating electrical and mechanical stimulation can reduce the parallel stiffness of the ECM and increase the serial stiffness, thereby enhancing the contractility of engineered skeletal muscle tissue [41]. Corresponding research findings indicate that proper exercise and training can stimulate the synthesis and degradation of collagen, optimizing the mechanical properties and load-bearing capacity of muscles. However, the lack of exercise gives rise to reduced collagen renewal, which affects the mechanical properties of muscles and potentially induces a reduction in muscularity and functionality [42]. During the ageing process, there is an augmentation of collagen I alongside a diminution of elastin fibers, culminating in heightened muscle rigidity and compromised adaptability [43]. This stiffening of the ECM impair the expansion and lineage commitment of MuSCs, thereby diminishing the regenerative potential of the muscle [44]. In sum, the status of collagen is closely associated with the structural and functional integrity of skeletal muscle. However, the dynamic changes in collagen architecture across various stages of injury and atrophy require further exploration. Developing tools that monitors the real-time change of ECM stiffness will facilitate timely clinical interventions. Additionally, future research should delve into how collagen architecture influences the lateral transmission of muscle contractile forces, particularly under different injury states. Such insights will aid in enhancing the efficacy of muscle functional recovery.

Proteoglycans in the ECM are instrumental in modulating diverse biological processes within skeletal muscle. Specifically, proteoglycans such as syndecan-4 and glypican-1 mediate the modulation of proliferation and differentiation processes in MuSCs [45]. Other ECM proteoglycans such as core proteoglycans, biglycan, and betaglycan weaken the binding of TGF-β to its receptor complexes, thereby indirectly promoting the differentiation of skeletal muscle [46]. Matrix Gla protein (MGP) exerts a regulatory effect on myogenic processes by inhibiting the activity of the myogenesis inhibitory factor myostatin, which may help promote muscle regeneration or treat muscle atrophy [47]. The impact of exercise on proteoglycans cannot be overlooked. Both acute and chronic exercise regimens are associated with an upregulation of proteoglycan expression, specifically highlighting the induction of serglycin, a novel exercise-responsive proteoglycan within skeletal muscle tissue. These exercise-mediated alterations in ECM and proteoglycan expression are likely to confer beneficial effects on muscle adaptability and regenerative potential [48]. Correspondingly, certain pathological conditions such as diabetes may negatively impact the ECM's makeup and functionality. In the diabetic mouse model, the transcriptional profiling of decorin and lumican is upregulated, which may interfere with normal muscle structure and function [49]. It is noteworthy that proteoglycans, through the entanglement and hydration of their glycosaminoglycan chains, in conjunction with complex fibrillar structures such as collagen and elastin, collectively determine the viscoelastic properties of the ECM [50]. The ECM's nonlinear viscoelastic behavior, including strain-hardening and stress relaxation, exert a beneficial effect on myogenesis. These properties facilitate the nuclear localization and activation of muscle regulatory factor (MRTF) through mechanotransduction processes [51]. Collagen and proteoglycans constitute two of the most abundant classes of substances within skeletal muscle ECM, whose quantity and composition exert significant impact on muscle regeneration and functional maintenance. The collagen fiber network provides mechanical strength and stability to skeletal muscle and regulates cellular behavior through interactions with cell surface receptors. Notably, compared to collagen, proteoglycans could also modulate cell signaling pathways and cell behavior, thereby exerting additional functions to collagen. Therefore, designing bioactive materials incorporating both collagen and functional proteoglycans could provide more comprehensive effects on skeletal muscle repair. Future studies are needed to further explore the how maunipulation in the types and compositions of collagen and proteoglycans affect muscle regeneration. Thus, provide instructions on the adjustment of the content of various components within the materials. Compared to the current used decellularized matrices, the new approach will achieve more precise therapeutic effects suitable for different stages of injury.

Integrins, as transmembrane receptors situated on the cell surface, are instrumental in orchestrating cell migration, proliferation, and tissue morphogenesis by facilitating cell adhesion and signal transduction via interactions with fibronectin, laminin and collagen [52]. Elevated levels of α7β1 integrins in skeletal muscle enhance myocyte physiological functions, while also protecting mice from exercise-induced muscle damage by negatively regulating mechanotransduction [53,54]. Further investigation has demonstrated that α7β1 integrins promote load-induced skeletal muscle growth through a mechanism independent of mTORC1 [55]. Additionally, α7β1 integrins can increase fiber hypertrophy and the synthesis of new fibers in skeletal muscle after eccentric exercise [56].

HA, a constituent of the ECM, manifests at relatively low concentrations within skeletal muscle, yet it exerts a pronounced influence on muscle tissue homeostasis. HA levels exhibits dynamic fluctuations following hypertrophic stimuli, thereby promoting the mobilization of muscle progenitor cells and preventing precocious myotube fusion [57]. Evidence also suggests that HA, through its reciprocal engagement with CD44, facilitates the migration and proliferation of myogenic progenitor [58]. HA also acts as a lubricant and viscoelastic shock absorber in muscles, regulating nociception and inflammation [59].

The composition of the ECM in its normal state is indispensable for maintaining muscle tissue integrity and executing its functions. Modulating the proportions of ECM components or enhancing their condition offers a potentially efficacious approach to the treatment of skeletal muscle pathologies.

## 5 The application of ECM in the treatment of skeletal muscle injury

Severe muscle injuries often encounter poor healing, posing significant challenges in clinical practice. Tissue engineering strategies have become a burgeoning field, holding significant potential for augmenting muscle regeneration. The following sections will provide a comprehensive examination of the advancements in ECM-based muscle repair materials, emphasizing the therapeutic potential of pure ECM materials, ECM composite materials, and ECM-mimicking materials. These innovative biomaterials harness the intrinsic bioactivity of the ECM to facilitate the reconstruction of functional muscle tissue, presenting a suite of effective solutions for skeletal muscle injury.

### 5.1 Pure ECM materials

Pure ECM materials refer to those extracted from natural tissues through decellularization techniques, preserving the original structure and bioactive components of the ECM. These materials serve as three-dimensional templates in tissue engineering to guide cellular interactions.

**Fig. 3:**
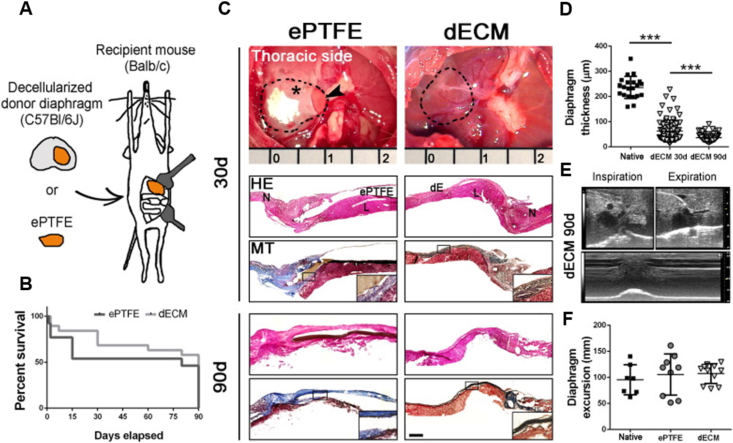
*In vivo* functional assessments, along with morphological and gross appearance evaluations, were conducted on diaphragms that had been subjected to treatment. (A) A schematic illustration depicts the retrieval and implantation process of both biological and synthetic mesh patches. (B) Surgical intervention outcomes for congenital diaphragmatic hernia. (C) A comparative assessment at 30 and 90 days post-surgery. (D) An analysis of diaphragmatic thickness is conducted. (E) Ultrasonographic imaging at 90 days postoperatively illustrates the diaphragmatic function. (F) Ultrasound echography was utilized to assess the mobility of the diaphragm following treatment. [Modified from Trevisan C et al. (64)].

In early studies, researchers implanted porcine small intestinal submucosa ECM (SIS-ECM) into the distal achilles tendon region of dogs and found that the ECM implant not only reduced the formation of scar tissue but also promoted the directional arrangement and maturation of muscle fibers. This discovery provided preliminary evidence for the application of ECM in muscle repair [60]. Subsequent studies further confirmed the reparative effects of SIS-ECM in rodent abdominal wall models. After six months, the implanted SIS-ECM was almost entirely replaced by islands and sheets of muscle, demonstrating that SIS-ECM not only promoted the morphological reconstruction of muscle tissue but also supported functional recovery [61]. Other than muscle tissue, ECM scaffolds have also shown potential in neural innervation. In prior investigations, the implantation of porcine ECM into rat abdominal wall reconstruction models and canine esophageal reconstruction models demonstrated the presence of neural tissues at the implant site, suggesting that ECM scaffolds could support the establishment of functional neural innervation [62]. Moreover, the ECM components of decellularized muscle scaffolds directly attract nerve axons, showing their inherent neurotrophic properties, which are necessary for the reconstitution of muscle function and the neuromuscular junctions [63]. Diaphragm-derived ECM is equally effective in repairing defects in rat latissimus dorsi muscles. This scaffold attracts various types of cells and induces angiogenesis and reinnervation (Fig. 3) [64]. These research results further confirm the versatility of ECM scaffolds in tissue repair. It is worth noting that the 3D architectural framework and biochemical milieu of skeletal muscle ECM are the principal determinants of muscle regeneration [65]. The distinct stages of the ECM also dictate its reparative efficacy; for instance, the early myogenic matrix can foster the myogenic differentiation of MuSCs by impeding myogenic inhibitory factors [66]. Corresponding research findings confirm that, compared to neonatal ECM, fetal ECM exhibits a more loosely arranged collagen network and larger pores, which can modulate the regenerative potential of tissues by dampening the signaling pathways implicated in inflammation and fibrosis [67].

Despite the promising muscle repair capabilities demonstrated by ECM derived from various sources, ECM derived from bladder has been shown to exacerbate immune responses and inhibit muscle regeneration. This suggests that GVHD may impact the reparative efficacy of the material, and it is essential to reduce the immunogenicity of the material during its fabrication. It is noteworthy that ECM-derived hydrogels treated with α-galactosidase can reduce xenoantigens, lower immunogenicity, and improve their safety in clinical applications [68].

The architectural attributes of the biological scaffold are determinant for the regenerative cascade of skeletal muscle. It has been clearly indicated that even in the absence of biochemical signals, tissue-specific morphological information can effectively initiate and guide the differentiation process of mesenchymal stem cells (MSCs) [69]. To better simulate the natural 3D structure of skeletal muscle, investigators have prepared ECM into bio-inks and used 3D printing technology to engineer skeletal muscle constructs with precisely controlled shapes, porosities, and microstructures. These ECM bio-inks not only provide a conducive cellular niche for cells, but also retain components such as agrin, further promoting the formation of neuromuscular junctions [70]. To further provide guidance clues for cell directional migration and spatial tissue arrangement, a study has developed ECM scaffolds with parallel microchannels and applied them to the repair of muscles, nerves, and blood vessels. These ECM scaffolds, by providing biochemical and topological clues, markedly enhance tissue regeneration, while simultaneously demonstrating excellent cellization and vascularization capabilities, as well as characteristics of regulating inflammatory responses [71]. By precisely controlling the ECM structure of cell deposition, ECM scaffolds maintain their structural anisotropy, providing continuous directional clues for myotube formation and effectively regulating the formation and arrangement of myotubes [72]. In addition, electrospinning technology is also used to prepare ECM scaffolds with adjustable physical and chemical properties. These scaffolds not only retain tissue-specific biochemical signals but also foster cellular engagement, expansion, and terminal myogenic differentiation. The fiber orientation and cross-linking degree of electrospun scaffolds significantly impact cell behavior, guiding the formation and arrangement of myotubes, providing robust support for skeletal muscle tissue engineering [73]. To summarized, preparation of pure ECM materials is not technically challenging. However, ECMs from different sources exhibit significant differences in immunogenicity and regenerative efficacy, which make it difficult for precise control of the molecular composition. Therefore, future research on pure ECM materials should focus on identifying more optimal ECM sources and reducing the immunogenicity of the materials. Additionally, detailed characterization of the specific roles of individual components within the ECM is required, which will facilitate the development of pure ECM materials with fewer side effects.

### 5.2 ECM composite materials

ECM composite materials mean the integration of ECM with other biomaterials or bioactive factors to form composite scaffolds with specific functions, thereby enhancing the effects of tissue repair and regeneration. Kasukonis et al. have utilized allogeneic decellularized skeletal muscle (DSM) scaffolds, either as standalone therapies or in conjunction with minced muscle (MM) autograft tissue, as a method for repairing VML. Studies have indicated that DSM scaffolds can provide a conducive environment for MuSCs, promoting muscle regeneration, and exhibit synergistic effects when used in conjunction with MM, enhancing the functional recovery of muscles [74]. The slurry is rich in myogenic cells and cytokines, greatly facilitating the repair of skeletal muscle. Combining ECM molecules secreted by muscle fibroblasts with autologous muscle slurry exhibits a significant improvement in muscle contractile torque and muscle mass recovery after VML injury [75]. Besides, a viable alternative approach is directly combine ECM with cells. Conconi et al. have combined ECM derived from the abdominal wall muscle with myoblasts, promoting angiogenesis and myotube formation in vivo, while the matrix without cell seeding is completely replaced by fibrous tissue [76]. Further testing with the addition of human adipose-derived stem cells (ASCs) in gastrocnemius muscle injury showed that the composite scaffold, when used with ASCs, can also provide a microenvironment conducive to muscle regeneration [77]. Meanwhile, ECM scaffolds derived from cardiac tissue, when combined with MSCs, can modulate the immune microenvironment by guiding macrophages to polarize towards the M2 phenotype, aiding in regenerative responses and muscle tissue repair [78]. Assessment of the outcomes of culturing myogenic differentiation cells with and without the use of bladder acellular matrix (BAM), was found that the tissue-engineered muscle regeneration constructs containing cellular components were more effective in promoting muscle functional recovery than BAM alone, highlighting the potential of ECM combined with cell therapy in tissue engineering [79]. However, some studies have shown that the combination of human muscle tissue-derived ECM with synthetic scaffolds did not perform well in promoting myotube formation, possibly due to changes in surface chemical properties caused by 1,6-hexanediamine [80]. This suggests that the formulation of engineered scaffolds needs to ensure the integrity of ECM's chemical and biological information.

Cell-composite scaffolds may encounter challenges related to cell survival. Incorporating cytokines into the ECM augments its cell-recruiting efficacy, thereby promoting a robust regenerative response. In a study utilizing decellularized skeletal muscle to fabricate 3D porous sponge-like ECM scaffolds and immobilizing stromal cell-derived factor-1 alpha (SDF-1α) within the scaffold. The findings indicate that these composite scaffolds significantly enhance the recruitment of multipotent stem cells and angiogenesis, thereby improving muscle regeneration outcomes [81]. ECM derived from skeletal muscle tissue, when combined with IGF-1, creates a muscle-specific microenvironment that promotes the mobilization and differentiation of MuSCs, thereby showing potential in the treatment of VML [82].

To simulate the natural structure of skeletal muscle, Kim et al. have modified ECM derived from porcine muscle tissue with methacrylate to enhance its mechanical stability as a bio-ink for 3D printing. The experimental outcomes indicated that the ECM-based 3D printed scaffolds promote the alignment and maturation of myotube cells by providing topological cues, which are crucial for myotube formation [83]. Mixing C2C12 mouse myoblasts with matrigel and fibrinogen, and integrating skeletal muscle and motor neurons to form solid muscle tissue rings in 3D printed hydrogel molds, also have good muscle-promoting effects [84]. Electrospinning technology has also simulated the nanostructure of skeletal muscle ECM. By modifying ECM with methacrylate to enhance its structural stability, making it more suitable for electrospinning, and combining it with poly(lactic-co-glycolic acid) (PLGA) microstructures to form composite scaffolds with multi-scale topological structures, these scaffolds can generate and maintain myotubes without myogenic medium conditions [85]. Combining bovine tail skeletal muscle ECM with Polycaprolactone (PCL) improved the mechanical stability of the scaffold, and by manufacturing aligned nanofiber scaffolds using electrospinning technology, mimicking the anatomical and biomechanical characteristics of natural muscle, which promoted the attachment, expansion, and differentiation of MuSCs [86].

ECM composite materials supplemented with pro-regenerative cells or growth factors exhibit superior tissue repair efficacy compared to pure ECM materials. Nevertheless, their capacity for recruiting and retaining cells remains limited and requires further optimization in future studies. Additionally, during the processing of ECM scaffolds, it is essential to maintain the integrity of the ECM's structural, chemical, and biological properties. Another important precaution is to avoid excessive cross-linking, which could compromise the biodegradability of the ECM or expose cryptic antigenic sites.

### 5.3 ECM-mimicking materials

ECM-mimicking materials are novel biomaterials fabricated through synthetic or biosynthetic methods, possessing structures and functions similar to those of the ECM. These materials are designed to emulate the microenvironment of the ECM to stimulate cellular functions and facilitate tissue repair. To overcome the issue of ECM sourcing and to reduce the GVHD caused by allogeneic ECM scaffolds, the extraction of effective ECM components as scaffold coatings can repair skeletal muscle to a certain extent. For instance, serum and fibrin coatings on scaffolds can promote the binding of growth factors, providing pivotal molecular cues for cells, which is critical for controlling MuSCs engraftment, migration, differentiation, specification, and apoptotic processes [87]. Further research has enhanced cell attachment and the provision of cytokine sustenance by covalently binding gelatin and heparin to the Alg-G-H matrix, effectively mimicking the biological functions of the ECM [88]. Laminin-111 (LM-111) is another component that has shown potential in ECM mimicry. By preparing it into a fibrin hydrogel encapsulating MSCs, Zahari et al. have found that it is capable of enhancing the survival rate and performance of MSCs within compromised muscle tissue, thus promoting the regeneration of skeletal muscle. Moreover, coating poly(methyl methacrylate) nanofiber scaffolds with laminin and collagen as biofunctional coatings also helps to promote the proliferation and migration of fibroblasts [89]. To better simulate the native ECM environment of cells, Besser et al. have combined gelatin and laminin through enzymatic cross-linking, controlling the biomechanical characteristics of the hydrogel by adjusting the concentration of gelatin, thereby facilitating the regeneration of muscle fiber [90]. In addition, a biosponge scaffold composed of pig skin gelatin solution, rat tail collagen I, and laminin-111, as a drug delivery system, can locally deliver the anti-fibrotic agent IDL-2965 to the VML injury area, effectively reducing fibrosis and promoting muscle recovery [91]. HA has also been used to create hydrogel scaffolds. By functionalizing HA hydrogels with ECM-derived laminin peptide IKVAV, researchers have promoted the activation and migration of MuSCs while inhibiting the excessive proliferation of fibroblasts [92]. Furthermore, the combination of electrical stimulation and laminin coating has been proven to enhance myotube formation, emphasizing the importance of considering the ECM environment and its combination with physical stimuli, such as electrical stimulation, in tissue engineering [93].

By imitating the arrangement and organization of collagen bundles in skeletal muscle tissue and designing nano-patterned substrates, an environment that promotes cell adhesion, migration, and differentiation can be created, thereby enhancing myogenic differentiation and maturation [94]. We fabricated a dual-crosslinked cryogel composed of methacrylated fucoidan and methacrylated gelatin (GelMA) to mimic the porous and interconnected structure of the ECM, which significantly enhanced vascularized skeletal muscle regeneration following VML and reduced collagen deposition [95]. Arab et al. have developed new tetrapeptide biomaterials that can self-assemble into a nanofiber 3D network, mimicking the natural collagen of the ECM and providing a 3D network structure similar to the natural environment for myoblasts, thus promoting the specific directional arrangement of cells [96]. Further research has treated fibrin microline scaffolds with etching technology, forming sub-micron grooves on the scaffold surface, which are consistent with the natural muscle ECM and help guide the alignment and differentiation of MuSCs, fostering the assembly of contractile muscle units [97]. In addition, 3D micro-patterned scaffolds prepared with ECM components, by replicating the cylindrical architecture of the muscle's basal lamina, guide muscle cells to align and form myotubes within microgrooves, which is not only conducive to muscle development and myogenesis but also helps maintain the integrity of the muscle [98]. To further simulate the natural structure of the ECM, Jana et al. have designed scaffolds containing nano-scale collagen fibers and aligned micro-scale basement membrane tracks, which help guide the alignment, migration, and differentiation of myoblasts, enhancing myotube development [99]. Notably, the application of 3D printing technology enables the fabrication of PLGA 3D-printed scaffolds that mimic the complex architecture of the ECM [100]. Electrospinning technology has also been used to prepare nanofiber scaffolds with ECM structural features. For instance, electrospun PCL-collagen I nanofibers can simulate the neat arrangement of muscle ECM, guiding the morphogenesis of myoblasts and promoting cell differentiation [101]. Besides, the incompletely porous fiber structure of electrospun poly(lactic-co-caprolactone) scaffold treated with a collagen coat gives rise to a niche that favors cell attachment and replicates the real environment of muscle tissue, accelerating the differentiation process of myoblasts and promoting the formation of muscle tissue [102]. Chitosan nanofiber mats accomplished by electrospinning can trigger the direction-directional growth and the shape-changing of C2C12 myoblasts in muscle tissue mimicking and remodeling [103]. In terms of promoting vascularization, PCL-Collagen I nanofiber scaffolds (PC) and scaffolds containing poly(ethylene oxide) sacrificial fibers produced by electrospinning have been used to simulate the ECM of skeletal muscle, showing excellent results [101]. These studies demonstrated that the ECM's geometry can be an important factor regulating the cellular activity, and therefore, tissue engineering of the skeletal muscle seems to be feasible by replicating the matrix geometry.

Conductivity has been recognized as a indispensable factor affecting myogenic differentiation. Recent research has pointed out that the use of nanoengineered conductive scaffolds enriched with extracellular Zn^2+^ ions can serve as an effective muscle differentiation factors, enhancing the response of myoblasts through synergistic stimulation. This strategy markedly elevates the proportion of myoblasts cultured compared to non-conductive surfaces. In addition, the collaborative impact further promotes the density, area, and diameter of myotubes, as well as the formation of multinucleated myotubes [104]. Further research has developed nano-composite hydrogel films with hierarchical structure and conductivity through microfluidic self-assembly technology, which can simulate the multi-scale hierarchical structure and electrical conductivity of the ECM. Mouse myoblasts C2C12 planted on these nano-composite fibrous hydrogel films show improved diffusion and enhanced myogenesis, indicating that this conductive hydrogel film provides a favorable microenvironment for muscle cells [105]. To simulate the electrophysiological properties of natural skeletal muscle, Zhang et al. have prepared conductive scaffolds by coating gold nanoparticles on PCL nanofiber nets, providing an electroactive interface for muscle cells. Using these conductive scaffolds to transmit electrical signals not only promotes the formation of myotubes but also accelerates the maturation process of myotubes. Electrical impulses enhance the development and contractile capabilities of myotubes by managing intracellular levels of ions and proteins, mirroring the intrinsic repair mechanisms of muscular tissue [106]. In addition, by adding conductive polymers to anionic polysaccharides, Srisuk et al. have successfully increased the electrical conductivity of hydrogels while maintaining their sponge-like structure [107]. Electrospinning and melt electrowriting technology are also used to manufacture 3D scaffolds with aligned nanofibers and micro-scale features. The scaffold's conductivity is enhanced by a gold nano-layer coating, providing electrical cues for muscle cells, further promoting the formation and maturation of myotubes [108].

ECM-mimicking materials, which can precisely control their structural and bioactive properties, represent a promising research direction (Table 1). However, several issues have been raised and should be taken into consideration. First of all, the characterization of ECM-mimicking materials is time-consuming and resource-intensive, lacking unified standards and guidelines. Secondly, the cost of ECM-mimicking materials are relatively high at this stage, particularly when 3D printing or electrospinning is required. Based on current research progress, it is challenging and far from satisfying for clinical translation. Additionally, skeletal muscle repair is a complex process involving the interplay of multiple factors, involving dynamic remodeling of the ECM, activation and differentiation of MuSCs, response of inflammatory cells, regulation of growth factors and cytokines, and regeneration of nerves and blood vessels. Therefore, when designing ECM-mimicking materials, it is essential to incorporate a variety of regenerative-promoting characteristics, not be limited to a structure similar to that of native ECM, appropriate mechanical properties, and efficient signaling functions.

**Table 1:** The application of ECM in the treatment of skeletal muscle injury.

Type of Material	Material Composition	Material Processing Method	Material Function	References
Pure ECM materials	Porcine; SIS-ECM	Decellularization;	Inducing vascularized and innervated skeletal muscle regeneration; Promoting the morphological reconstruction of muscle tissue; Supporting functional recovery	[60,61]
	Porcine; Urinary Bladder Matrix (UBM)-ECM	Decellularization;	Supporting nerve regeneration; Promoting muscle tissue regeneration.	[62]
	Skeletal muscle ECM derived from rat hindlimb	Decellularization;	Supporting neuronal axon growth; Retaining neuroregeneration-related proteins; Intrinsic neurotrophic properties.	[63]
	Diaphragm tissue ECM; Expanded polytetrafluoroethylene (ePTFE)	Decellularization;	Promoting angiogenesis; attracting nerve regeneration; Supporting muscle regeneration and functional recovery.	[64]
	ECM derived from C2C12 myoblasts	Decellularization;	Suppressing cell growth; Promoting myotube formation.	[66]
	Fetal dermis; subcutaneous tissue; Muscle ECM derived from rabbits and rats	Decellularization;	Promoting myocyte ingrowth and myotube formation; Suppressing expression of inflammatory and fibrotic genes; Supporting neovascularization.	[67]
	Minced Muscle Graft; Urinary Bladder ECM	Micronized	Promoting de novo muscle fiber regeneration and functional recovery	[70]
	ECM induced by PCL microfiber templates	Decellularization;	Supporting vascularization and immunomodulation; Regenerating skeletal muscle, nerve, and artery tissues.	[71]
	ECM derived from rabbit skeletal muscle	Decellularization; Electrospinning	Controlling myotube formation and alignment; Supporting cell-mediated remodeling of the dECM substrate.	[73]
ECM composite materials	Skeletal muscle ECM; Minced muscle; Both derived from rats	Decellularization;	Increasing muscle contractile force recovery and muscle mass recovery; Reducing fibrotic response at the repair site.	[74]
	Combining ECM molecules secreted by muscle fibroblasts with autologous muscle slurry	Decellularization;	Improving the muscle contractile torque and muscle mass recovery.	[75]
	Abdominal wall muscle ECM; Myoblasts; Both derived from rats	Decellularization;	Promoting angiogenesis and myotube formation	[76]
	Skeletal muscle ECM derived from rats; Human Adipose-Derived Stromal Cells;	Decellularization;	Supporting de novo muscle fiber formation and improving muscle regeneration; Regulating RAGE and p38 MAPK signaling	[77]
	ECM derived from porcine heart tissue; MSCs	Decellularization;	Regulating macrophage polarization toward the M2 phenotype; Improving tissue repair and functional recovery	[78]
	Bladder ECM derived from porcine; Muscle-derived cells	Decellularization;	Promoting skeletal muscle regeneration and improving functional recovery	[79]
	Rat skeletal muscle ECM; SDF-1α	Decellularization;	Promoting angiogenesis and muscle progenitor recruitment; Improving skeletal muscle regeneration	[81]
	Rabbit skeletal muscle ECM; IGF-1	Decellularization;	Promoting skeletal muscle regeneration; Supporting MuSCs adhesion, proliferation, and differentiation	[82]
	Porcine skeletal muscle ECM; Poly(vinyl alcohol) (PVA)	Decellularization; 3D printing	Promoting alignment and differentiation of skeletal muscle cells; Supporting efficient myotube formation	[83]
	C2C12 mouse myoblasts; Matrigel; Fibrinogen; Skeletal muscle; motor neurons;	3D printing	Mimicking NMJ function; Enabling muscle contraction via chemical stimulation	[84]
	Porcine skeletal muscle; PLGA; GelMA	Decellularization; Electrospinning; 3D printing	Promoting orientation and maturation of human muscle progenitor cells; Supporting myotube formation	[85]
	Bovine tail skeletal muscle ECM; PCL	Decellularization; Electrospinning	Simulating muscle fiber alignment; Providing mechanical support and topographical cues for cell growth	[86]
ECM-mimicking materials	Polystyrene sub-micron fibers; Serum; Fibrin; FGF-2; BMP-2	Spinneret-based Tunable Engineered Parameters	Mimicking ECM structure and controlling cell alignment; Controlling muscle cell differentiation	[87]
	Porcine skeletal muscle ECM; Gelatin; Heparin; Alginate	Chemically modified	Mimicking the natural microenvironment of skeletal muscle cells to support proliferation, differentiation, and myotube formation	[88]
	Poly(Methyl Methacrylate) (PMMA); Collagen; Laminin; Genipin;	Electrospinning	PMMA serves as a 3D scaffold for cell attachment and growth; Collagen promotes fibroblast proliferation and migration; Laminin promotes myoblast proliferation and migration, and enriches myoblast population; Genipin enhances protein adsorption on the scaffold surface.	[89]
	Laminin; Gelatin	Mixing; Crosslinking	Mimicking the composition and mechanical properties of the ECM; Supporting the culture of neurons, Schwann cells and skeletal muscle cells	[90]
	Gelatin; Collagen; Laminin; Anti-fibrotic agent (IDL-2965)	Crosslinking; Electrostatic interactions	Inhibiting fibrotic tissue deposition; Supporting muscle regeneration and functional recovery; Facilitating force transmission.	[91]
	HA; Poly(ethylene glycol diacrylate) (PEGDA); ECM-derived peptides	Crosslinking; Covalent bonding	Mimicking the regenerative environment; Inhibiting excessive fibroblast proliferation; Reducing fibrotic tissue formation;	[92]
	Polyacrylamide (PA) gels; Matrigel; Laminin; Poly-D-lysine	Crosslinking;	Mimicking the in vivo niche of skeletal muscle cells	[93]
	Polyurethane Acrylate (PUA); Gold]; Fibronectin	Capillary force lithography; Electron beam evaporation	Mimicking the nanotopographical features of skeletal muscle ECM; Enhancing myogenic differentiation and maturation; Promoting myotube alignment and growth.	[94]
	Methacrylated fucoidan; GelMA	Freezing; Crosslinking	Promoting vascularized skeletal muscle regeneration; Reducing collagen deposition; Improving mitochondrial energy metabolism	[95]
	Ultrashort self-assembling peptides (CH-01, CH-02), alginate-gelatin blend	Self-assembly	Mimicking the ECM nanofibrous structure to support cell growth and differentiation; Providing a 3D culture environment to promote myoblast alignment and proliferation	[96]
	Fibrinogen; Thrombin	3D printing	Mimicking the morphology of native muscle tissue; Enhancing myoblast alignment and filamentous actin stress fiber organization; promoting functional muscle tissue regeneration.	[97]
	Type I Collagen	N/A	Mimicking the structure of skeletal muscle basement membrane to promote alignment of myoblasts and formation of multi-layered muscle bundles.	[98]
	Chitosan; PCL; Collagen Type I	Electrospinning	Mimicking the nanoscale and microscale structures of the skeletal muscle ECM to promote myoblast alignment and differentiation	[99]
	PLGA	3D printing	Mimicking the complex architecture of the ECM	[100]
	PCL; Collagen I; Polyethylene oxide; Fibrin gel	Electrospinning	Mimicking the ECM of skeletal muscle to support cell growth and differentiation; Promoting neovascularization and neurotization by optimizing fiber architecture.	[101]
	Poly(L-lactide-co-ε-caprolactone) (PLCL); Type I collagen;	Electrospinning	Mimicking the nanoscale structure of skeletal muscle ECM to promote adhesion, proliferation, and differentiation of C2C12 myoblasts.	[102]
	Chitosan; Polyethylene oxide (PEO); Dibasic Sodium Phosphate (DSP)	Electrospinning; Crosslinking	Mimicking the ECM structure with nanofibrous mats for soft tissue regeneration; Providing mechanical properties similar to soft tissues	[103]
	PCL; Graphene nanosheets; Zinc ions	Solvent evaporation	Providing surface conductivity similar to that of skeletal muscle tissue; Promoting cell proliferation and differentiation via the PI3K/Akt signaling pathway	[104]
	Chitosan; Gellan gum; Graphene; Fibrous hydrogel	Electrostatic self-assembly;	Recapitulating the multiscale hierarchy and electrical conductivity of native skeletal muscle tissue; Enhancing cell adhesion, spreading, and myotube formation	[105]
	PCL; Gold nanoparticles	Electrospinning	Recapitulating the anisotropic structure and electrical conductivity of skeletal muscle tissue; Promoting alignment, differentiation, and elongation of H9c2 cells into myotubes	[106]
	Gellan Gum; Polyaniline	Crosslinking	Recapitulating the porous structure and electrical conductivity of the skeletal muscle ECM; Supporting cell adhesion, proliferation, and differentiation; promoting fusion of C2C12 myoblasts into myotubes	[107]
	PCL; Gold nanoparticles (AuNPs); Gellan Gum	Electrospinning	Recapitulating the multiscale hierarchical structure and electrical conductivity of skeletal muscle tissue; Promoting alignment, fusion, and elongation/maturation of H9c2 cells into myotubes	[108]

## 6 The application of ECM in the treatment of skeletal muscle atrophy

Muscle wasting is a weakening condition where muscle mass and strength slowly disappear. It's a big challenge within the realm of regenerative medicine. Lots of studies have been conducted to explore whether ECM components can help muscle recovery. This process is highly analogous to the natural regeneration of muscle tissue. In this segment, we explore the complex interplay between the ECM and the muscle tissue, underscoring the significance of therapies derived from the ECM, particularly in treating muscle atrophy. We will traverse through the latest research findings that highlight the utility of ECM components in modulating muscle microenvironment, cytokine expression, and overall muscle function. The subsequent discussion will illuminate the functions of diverse proteins within the ECM and the potential for their precise control to forge new therapeutic approaches.

### 6.1 Treatment of muscular atrophy with ECM components

Earlier studies have shown that injecting ECM sourced from skeletal muscle into denervated muscle can effectively boost muscle function and walking patterns. This treatment also decreases the levels of inflammatory cytokines, which is closely linked to the strengthening of muscle power. [109]. Subsequent investigations have highlighted the augmented therapeutic potential of ECM, when integrated with HA, in creating a biological scaffold that is conducive to muscle repair. This scaffold not only recruits regenerative immune cells but also modulates the polarization of macrophages, and locally delivers myostatin inhibitors to enhance their bioactivity, promoting muscle regeneration in the DMD animal model [110].

In exploring the therapeutic properties of ECM components, LM-111, a protein of the ECM, has demonstrated its ability to ameliorate muscle tissue conditions in mdx mice. This improvement is attributed to the enhanced production of the α7β1 integrin. Systemic delivery of LM-111 to the skeletal muscles through intraperitoneal injection distributes it across multiple muscle tissues, offering new possibilities for systemic treatment of DMD [111]. Nevertheless, the presence of collagen could potentially disrupt the interaction between LM-111 and the α7β1 integrin. This interference might impact the muscle's ability to adapt to mechanical stress [112].

Non-glycosylated biglycan (NG biglycan) is a component of the ECM. In DMD, the function of NG biglycan may be compromised, resulting in the deterioration of muscle fibers and the development of fibrosis. By employing genetic manipulation and pharmaceutical strategies to either restore or emulate the typical functioning of NG biglycan, it may be possible to alleviate the muscle-related issues experienced by individuals with DMD. For instance, systemic administration of recombinant NG biglycan in dystrophic mice elevate the levels of utrophin and improve muscle health and function [113].

MGP, as part of the ECM, regulates the muscle cells by interacting with other ECM proteins such as myostatin. The regulatory effect of MGP on myostatin may interfere with the binding of myostatin to activin receptor type IIB, thereby affecting the TGF-β/SMAD signaling pathway, which is a essential regulatory mechanism in muscle development. These research findings indicate the capacity of ECM in muscle development and disease treatment, especially in muscle diseases such as muscular atrophy [47].

Currently, the direct application of ECM components in the treatment of skeletal muscle atrophy remains relatively underexplored. Although some studies have demonstrated potential therapeutic effects, the precise efficacy and underlying mechanisms require further investigation to be definitively established.

### 6.2 Modulating ECM components for the treatment of muscular atrophy

The buildup of collagen in skeletal muscle is a characteristic sign of the aging process. This accumulation correlates with a decline in muscle performance and a simultaneous decrease in the muscle's capacity for self-repair. By modulating macrophages and MuSCs in the muscle, the remodeling of the ECM, including the synthesis and degradation of collagen, may be indirectly affected, thereby improving muscle mass and function [114]. The deficiency of collagen VI has been linked to the development of congenital muscular dystrophy. Notably, adipose-derived stromal cells (ADSCs) exhibit the propensity to abundantly produce and secrete collagen VI. By implanting ADSCs into the skeletal muscle of mice afflicted with Congenital Muscular Dystrophy (CMD), a significant enhancement of collagen VI levels within the muscle tissue was achieved, thereby facilitating muscle repair [115]. Another study employs gapmer antisense oligonucleotides to silence mutated transcripts, thereby augmenting the deposition of collagen VI protein, representing a potential therapeutic strategy for collagen VI-related CMD [116]. In summary, different types of collagens exert distinct roles during muscle atrophy. Future research investigating the functions of various collagen types is warranted to facilitate more precise modulation of collagen levels at sites of atrophy and enhance therapeutic outcomes for muscle atrophy.

**Fig. 4:**
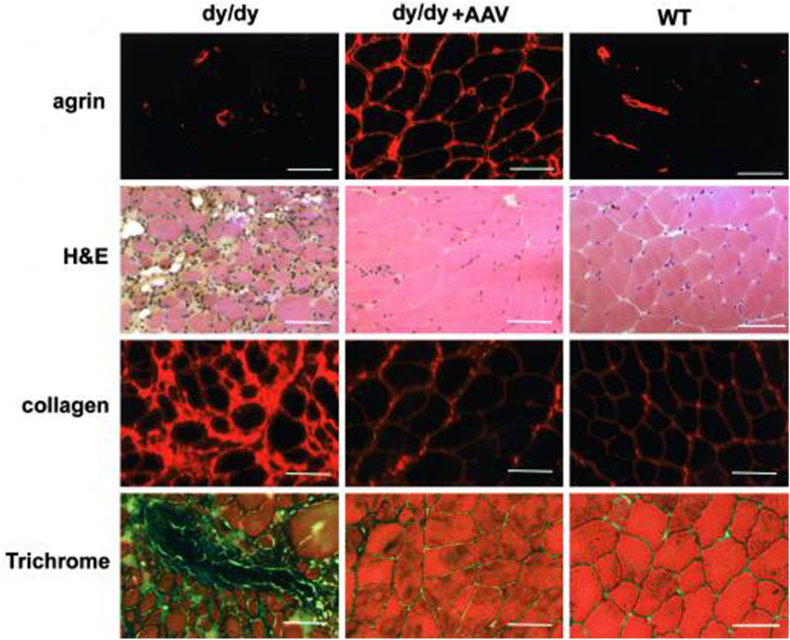
The targeted enhancement of miniagrin gene expression was investigated for its potential to mitigate the pathological features in the dystrophic muscles of dy/dy mice. Histological assessments were conducted on the muscle harvested two months post-injection, comparing untreated dy/dy mice, those treated with the AAV2-miniagrin vector (Center panel), and wild-type littermates. The muscles were analyzed using immunofluorescence (IF) staining for agrin and collagen III, as well as hematoxylin and eosin (H&E) and Masson's Trichrome staining to evaluate tissue morphology. The results demonstrated that elevated miniagrin levels were associated with significant amelioration of muscle histopathology and a reduction in fibrotic deposition within the dystrophic muscle tissue. (Scale bars: 100 μm) [Modified from Qiao C et al. (118), Copyright (2005) National Academy of Sciences, U.S.A.].

Laminin-α2 is a vital protein found within the muscle's ECM. Genetic alterations affecting the laminin-α2 gene can lead to the development of CMD. In laminin-α2-deficient congenital muscular dystrophy 1A (MDC1A), the absence of the laminin-α2 chain impairs the connection between myofibers and the ECM, leading to muscle degeneration. Findings from various studies suggest that miniagrin, through its binding to laminins, can restore the mechanical stability of muscle fibers, reduce fiber breakage and fibrosis, and ameliorate the pathological characteristics of muscle tissue and motor performance in MDC1A mouse models (Fig. 4) [[117], [118], [119]]. Additionally, some studies have restored the function of laminin-α2 in the muscle ECM through gene therapy approaches, thereby stabilizing muscle fibers and preventing the progression of muscular diseases [120,121]. Laminin, through its association with the dystrophin glycoprotein complex (DGC), links to the cytoskeleton, thereby helping to maintain the stability and integrity of muscle fibers. In pathological states of muscle atrophy, the membrane localization of the DGC is affected, potentially culminating in muscle fiber membrane instability and a subsequent deterioration of muscle function. Studies have explored enhancing the stability of the DGC by phosphorylating dystrophin S3059, which may reinforce the interaction between muscle fibers and laminin, potentially providing a new strategy for treating muscle wasting [122]. Overexpression of sarcospan can also improve the binding of the DGC to laminin, thereby enhancing the stability of the sarcolemma of muscle fibers and reducing muscle pathology [123]. To summarize, activation of laminin-α2 directly or indirectly has proven to be effective strategies for treating muscle atrophy. Thus, developing small-molecule drugs targeting laminin-α2 requires further investigation to improve their specificity and delivery efficiency. Besides, utilization of CRISPR/Cas9 gene-editing technology to repair laminin-α2 gene mutations hold great potentials for clinical application. This approach, if achieved with high safety, efficacy, and durability, could directly target and correct the laminin-α2 gene mutations that cause CMD, thereby addressing the root cause of the disease.

Fibrinogen deposition in the ECM is considered a crucial factor in the progression of DMD pathology as it associates with the activation of inflammatory cells and impaired muscle tissue regeneration. Genetic or pharmacological interventions that reduce fibrinogen deposition in the ECM can reduce inflammation and improve muscle regeneration, which may provide a new strategy for treating DMD [124].

Versican is a transitional ECM protein that regulates muscle development and regeneration, and its expression is upregulated within the muscular tissue of mdx mice, associated with impaired muscle regeneration. Glucocorticoids facilitate myogenic differentiation by impacting the formation and degradation of versican, indicating that glucocorticoids may ameliorate the disease symptoms of DMD by regulating the composition of the ECM [125]. A disintegrin and metalloproteinase with thrombospondin motifs-5 (ADAMTS-5) is an enzyme that degrades versican and is upregulated in the mdx mouse model, co-localizing with injuryed muscle. The blockade of ADAMTS-5 improves muscle strength in fast muscle fibers, indicating that the modulation of the ECM may have a positive effect on DMD treatment. Research suggests that ADAMTS-5 and versican may have therapeutic potential in DMD pathology [126]. ADAMTS1 is involved in the pathological process of muscle through its interaction with ECM proteins, and its ability to cleave ECM components may affect muscle regeneration and fibrosis. Anti-ADAMTS1 treatment alleviates abnormal ECM deposition in mdx mice by modulating the transcript levels and deposition of ECM-related proteins, improving myofiber integrity and muscle performance, indicating adjusting the ECM could offer a therapeutic strategy for DMD [127]. Notably, the use of glucocorticoids, ADAMTS-5 inhibitors, and ADAMTS1 inhibitors requires further investigation to mitigate side effects associated with nonspecific inhibition.

Latent Transforming Growth Factor-Beta Binding Protein 4 (LTBP4) modulates the development of muscle disease by regulating the release and activity of TGF-β. The genetic polymorphism of LTBP4 influences the activity of TGF-β, which in turn impacts the severity of muscle disease. In therapeutic strategies, modulating factors related to the ECM, such as LTBP4 and TGF-β, can be a method to improve muscle disease [128]. Anti-LTBP4 antibodies target the hinge region of LTBP4, reducing the hydrolysis of LTBP4, thereby stabilizing the muscle membrane and reducing muscle fibrosis, indicating that the modulation of ECM components can be a strategy for treating muscle disease [129].

Biglycan is a leucine-rich proteoglycan that can bind to multiple proteins, including α-dystroglycan, α-sarcoglycan, and c-sarcoglycan. These sarcoglycans collectively form the DGC on the muscle fiber membrane. Dysfunction of this complex is associated with a spectrum of muscular disorders. By delivering the human biglycan gene hBGN to mdx mice through the rAAV8 viral vector, Ito et al. detected an elevation in biglycan expression and its anchoring role in muscle fibers, improving muscle pathological symptoms by upregulating the expression of DGC proteins. Even with low transduction efficiency, the anchoring strategy of ECM proteins can significantly improve muscle function and pathological state in mdx mice, providing a new potential treatment for DMD [130].

MMPs are involved in controlling muscle fiber regeneration and inflammatory responses by degrading collagen and other matrix proteins in the ECM. In the context of DMD, MMP-9 exhibits heightened expression and enhanced catalytic activity. The suppression of MMP-9 activity has been demonstrated to foster muscle fiber regeneration, attributable to the attenuation of inflammatory responses and fibrotic processes, concurrently augmenting the efficacy of muscle progenitor cell engraftment within compromised muscle regions [131]. Within the muscular tissue of mdx mice, inhibiting the activity of MMP-9 can further reduce ECM degradation, improve the stability of muscle fibers, and thus help improve muscle function and delay disease progression in DMD patients, which is of potential significance for their treatment [132]. MMP-10 is also instrumental in preserving homeostatic equilibrium of the muscle ECM. The reduction in MMP-10 activity leads to impaired ECM remodeling, which in turn impairs the interaction between MuSCs and the ECM, resulting in cellular senescence and decreased muscle repair capacity [133]. Additionally, MMP-10 potentially modulates muscle repair via the VEGF/Akt signaling cascade, which is central to the functionality of satellite cells and the process of angiogenesis [134]. Therefore, modulating the levels of MMP-10 can positively facilitate muscle regeneration and repair. Besides MMP-9 and MMP-10, MMP-1 also show potential therapeutic effects in muscle diseases. For example, Wharton's jelly MSCs secrete MMP-1, which can reduce the accumulation of ECM components, particularly by lowering the levels of fibronectin, thereby reducing muscle fibrosis and improving muscle function [135]. These therapeutic approaches, which modulate the activity of MMPs or leverage MMPs secreted by stem cells, have demonstrated potential therapeutic value in ameliorating muscle fibrosis and promoting regeneration. However, further research is needed to optimize their efficacy and safety. For instance, the dynamic changes of MMP-9 and MMP-10 across different stages of atrophy could be further explored, and more specific inhibitors or activators with less off-target effects could be developed to achieve precise therapeutic interventions.

Integrins, serving as receptors for fibronectin, laminin, and collagen, mediate the connection between cells and the ECM, as well as engage in cellular signal transduction and a variety of biological processes. The α7β1 integrin, serving as a receptor for laminin, may aid in compensating for the structural deficiencies arising from the absence of dystrophin, thereby potentially mitigating the progression of DMD [136]. Research has revealed that overexpressing α7 integrin in the muscles of DMD mice provides significant protection against force loss induced by eccentric contractions. Post-treatment, expanded muscle fiber dimensions in the mice confirms the therapeutic efficacy of α7 integrin in treating skeletal muscle atrophy [137]. Corresponding research has illustrated that increasing the expression of the viral-mediated b1D chain can promote the interaction between α7β1 integrin and laminin, thereby enhancing the linkage of myofibers to the ECM and reducing muscle damage [138]. SU9516, a small molecule capable of increasing the expression of α7β1 integrin, may function by improving the connection between the ECM and muscle cells [139]. Therapeutic approaches based on α7β1 integrin have demonstrated significant protective effects in improving muscle function in mouse models of DMD. However, their clinical application will be limited by potential risks to cardiac function due to lack of specific drug delivery system. Future research is recommended to further optimize drug delivery systems for α7β1 integrin-related therapies, enhancing their targeting specificity and delivery efficiency in skeletal muscle.

In summary, the aforementioned studies have collectively validated the feasibility of treating skeletal muscle atrophy by modulating the ECM components (Table 2). These findings enhance our understanding of ECM's significance in muscle health and disease, while also provide a scientific foundation for devising innovative treatment approaches.

**Table 2:** Treating skeletal muscle atrophy by modulating ECM components.

Myopathies	Modulated ECM components	Modulation methods	References
Sarcopenia	Collagen IV	Reducing collagen IV levels	[114]
Collagen VI-related CMD	Collagen IV	Elevating collagen IV levels	[115]
	Collagen IV	Enhancing the deposition of functional collagen IV	[116]
MDC1A	Laminin-α2	Restoring the function of laminin-α2	[[117], [118], [119]]
	Laminin-α2	Restoring the function of laminin-α2	[120,121]
DMD	Fibrinogen	Reducing fibrinogen deposition	[124]
	Versican	Inhibiting the synthesis of versican	[125]
	Versican	Suppressing the expression of versican	[126]
	Versican	Suppressing the expression of versican	[127]
	LTBP4	Inhibiting the activation of TGFβ	[129]
	Biglycan	Upregulating biglycan levels	[130]
	MMP-9	Reducing MMP-9 levels.	[132]
	MMP-10	Upregulating MMP-10 levels	[134]
	MMP-1	Upregulating MMP-1 levels	[135]
	α7 integrin	Overexpressing human α7 integrin	[137]
	α7β1 integrin	Enhancing the expression of α7β1 integrin	[136]
	α7β1 integrin; laminin	Enhancing the expression of α7β1 integrin and laminin	[138]
	α7β1 integrin	Promoting the expression of α7β1 integrin	[139]

## 7 Summary and prospects

The ECM of skeletal muscle, comprising macromolecules secreted by cells into their external environment, is essential for cellular signal transduction and the tissue response to injury and disease [140]. Modulation of the ECM's constituent elements, structural attributes, and biological functions can enhance the reconstitution and mending of muscle fibers.

Currently, the therapies for skeletal muscle injury involving the ECM primarily utilize ECM-based scaffolds. These scaffolds retain or mimic the original ECM environment, effectively facilitating the restoration of injured muscles [12]. The physical form, chemical composition, and integration of bioactive factors of ECM scaffolds offer a multifunctional repair platform for tissue engineering. In the therapeutic approach to muscle wasting, the modulation of ECM components, such as laminin, biglycan, and MMPs, has shown potential to improve muscle pathology and promote functional recovery. However, there are limitations in the current understanding and application. Firstly, there are issues regarding the immune response to scaffolds and their integration with host tissues. The initial cause of immune reactions is due to the residual antigenic components within the ECM, which subsquently activate the host immune system. Although multiple studies have supported the biocompatibility of ECM scaffolds in tissue engineering, those derived from xenogeneic or allogeneic sources could still elicit immune responses, leading to graft rejection or inflammatory reactions that inhibit muscle regeneration [68]. Moreover, the long-term stability of ECM and its integration mechanisms with host tissues remain less understood, which restricts its widespread clinical application. Secondly, the composition of ECM is highly complex, encompassing collagen, elastin, glycosaminoglycans, and other components. The extraction and purification processes of these components are difficult to standardize. Resulting in inconsistent therapeutic outcomes due to the heterogeneity in compositions and structures of ECMs from different sources [60,61]. Additionally, during the extraction and processing of ECM, some bioactive components are lost or denatured, leading to impaired functionality [80]. Raising the question that how to select appropriate sources of ECM and maximize the retention of its bioactivity during processing.

In terms of modulating ECM components for the treatment of skeletal muscle injury or atrophy, the main challenge is the lack of specificity. For example, in current DMD therapies, although inhibiting MMP-9 can improve muscle fiber regeneration, leakage of local inhibition may lead to fibrosis in other tissues, causing both inconsistent therapeutic effects and potential side effects [131]. Furthermore, the long-term stability and dynamic equilibrium of ECM components should be taken in to consideration in advance. For instance, excessive deposition of collagen can cause muscle fibrosis, while insufficient degradation can impair muscle regeneration [38]. However, current therapeutic strategies still have a long way to go to achieve this balance.

In future studies, more precise ECM modulation strategies should be developed, especially those through more accurate and personalized ways. For instance, employing gene-editing technology or small-molecule drugs targeting specific ECM components could reduce off-target effects. Additionally, a deeper understanding of the dynamic equilibrium mechanisms of ECM components is warranted. The development of biosensors or smart materials capable of real-time monitoring and regulation of ECM components would be highly advantageous. For example, intelligent ECM scaffolds that could respond to muscle injury signals and automatically regulate collagen synthesis and degradation represents a highly promising research direction. In the treatment of skeletal muscle atrophy, the underlying pathological mechanisms may vary among different patients. Therefore, further research is needed to diagnose the etiology of atrophy, thereby enabling the design of personalized ECM modulation strategies based on the pathological features and disease types of individual patients. Moreover, minimizing immune responses elicited by ECM components through surface modification or immune modulation strategies is essential for the clinical translation and application of these therapeutic approaches. Through a deep understanding of the biological characteristics of ECM and optimizing its therapeutic strategies, we can look forward to the emergence of more ECM-based treatments.

## Declaration of competing interest

The authors declare that they have no known competing financial interests or personal relationships that could have appeared to influence the work reported in this paper.

## References

[1] Golebiowska A.A., Intravaia J.T., Sathe V.M., Kumbar S.G., Nukavarapu S.P. (2023). Decellularized extracellular matrix biomaterials for regenerative therapies: advances, challenges and clinical prospects. Bioact Mater.

[2] Ahmad K., Shaikh S., Ahmad S.S., Lee E.J., Choi I. (2020). Cross-talk between extracellular matrix and skeletal muscle: implications for myopathies. Front Pharmacol.

[3] Ahmad K., Shaikh S., Chun H.J., Ali S., Lim J.H., Ahmad S.S. (2023). Extracellular matrix: the critical contributor to skeletal muscle regeneration-a comprehensive review. Inflamm Regen.

[4] Cai L., Shi L., Peng Z., Sun Y., Chen J. (2023). Ageing of skeletal muscle extracellular matrix and mitochondria: finding a potential link. Ann Med.

[5] Tamaki T. (2020). Biomedical applications of muscle-derived stem cells: from bench to bedside. Expet Opin Biol Ther.

[6] Mukund K., Subramaniam S. (2020). Skeletal muscle: a review of molecular structure and function, in health and disease. Wiley Interdiscip Rev Syst Biol Med.

[7] Grasman J.M., Zayas M.J., Page R.L., Pins G.D. (2015). Biomimetic scaffolds for regeneration of volumetric muscle loss in skeletal muscle injuries. Acta Biomater.

[8] Rahman F.A., Baechler B.L., Quadrilatero J. (2024). Key considerations for investigating and interpreting autophagy in skeletal muscle. Autophagy.

[9] Qaisar R., Bhaskaran S., Van Remmen H. (2016). Muscle fiber type diversification during exercise and regeneration. Free Radic Biol Med.

[10] Wang Y., Lu J., Liu Y. (2022). Skeletal muscle regeneration in cardiotoxin-induced muscle injury models. Int J Mol Sci.

[11] Duda G.N., Taylor W.R., Winkler T., Matziolis G., Heller M.O., Haas N.P. (2008). Biomechanical, microvascular, and cellular factors promote muscle and bone regeneration. Exerc Sport Sci Rev.

[12] Philips C., Terrie L., Thorrez L. (2022). Decellularized skeletal muscle: a versatile biomaterial in tissue engineering and regenerative medicine. Biomaterials.

[13] Chazaud B. (2020). Inflammation and skeletal muscle regeneration: leave it to the macrophages!. Trends Immunol.

[14] Jiang H., Liu B., Lin J., Xue T., Han Y., Lu C. (2024). MuSCs and IPCs: roles in skeletal muscle homeostasis, aging and injury. Cell Mol Life Sci.

[15] Yin H., Price F., Rudnicki M.A. (2013). Satellite cells and the muscle stem cell niche. Physiol Rev.

[16] Bagalad B.S., Mohan Kumar K.P., Puneeth H.K. (2017). Myofibroblasts: master of disguise. J Oral Maxillofac Pathol.

[17] Mofarrahi M., McClung J.M., Kontos C.D., Davis E.C., Tappuni B., Moroz N. (2015). Angiopoietin-1 enhances skeletal muscle regeneration in mice. Am J Physiol Regul Integr Comp Physiol.

[18] Zhang X., Hu F., Li J., Chen L., Mao Y.F., Li Q.B. (2024). IGF-1 inhibits inflammation and accelerates angiogenesis via Ras/PI3K/IKK/NF-κB signaling pathways to promote wound healing. Eur J Pharmaceut Sci.

[19] Rodgers J.T., Schroeder M.D., Ma C., Rando T.A. (2017). HGFA is an injury-regulated systemic factor that induces the transition of stem cells into GAlert. Cell Rep.

[20] Verma M., Asakura Y., Murakonda B.S.R., Pengo T., Latroche C., Chazaud B. (2018). Muscle satellite cell cross-talk with a vascular niche maintains quiescence via VEGF and notch signaling. Cell Stem Cell.

[21] Gordon T. (2020). Peripheral nerve regeneration and muscle reinnervation. Int J Mol Sci.

[22] Sartori R., Romanello V., Sandri M. (2021). Mechanisms of muscle atrophy and hypertrophy: implications in health and disease. Nat Commun.

[23] Sayer A.A., Cooper R., Arai H., Cawthon P.M., Ntsama Essomba M.J., Fielding R.A. (2024). Sarcopenia. Nat Rev Dis Primers.

[24] Shen Y., Zhang C., Dai C., Zhang Y., Wang K., Gao Z. (2024). Nutritional strategies for muscle atrophy: current evidence and underlying mechanisms. Mol Nutr Food Res.

[25] Schiaffino S., Mammucari C. (2011). Regulation of skeletal muscle growth by the IGF1-Akt/PKB pathway: insights from genetic models. Skeletal Muscle.

[26] Sartori R., Gregorevic P., Sandri M. (2014). TGFβ and BMP signaling in skeletal muscle: potential significance for muscle-related disease. Trends Endocrinol Metabol.

[27] Chen Z., Li L., Wu W., Liu Z., Huang Y., Yang L. (2020). Exercise protects proliferative muscle satellite cells against exhaustion via the Igfbp7-Akt-mTOR axis. Theranostics.

[28] Blazev R., Carl C.S., Ng Y.K., Molendijk J., Voldstedlund C.T., Zhao Y. (2022). Phosphoproteomics of three exercise modalities identifies canonical signaling and C18ORF25 as an AMPK substrate regulating skeletal muscle function. Cell Metab.

[29] Quattrocelli M., Wintzinger M., Miz K., Levine D.C., Peek C.B., Bass J. (2022). Muscle mitochondrial remodeling by intermittent glucocorticoid drugs requires an intact circadian clock and muscle PGC1α. Sci Adv.

[30] Yin L., Li N., Jia W., Wang N., Liang M., Yang X. (2021). Skeletal muscle atrophy: from mechanisms to treatments. Pharmacol Res.

[31] Gallagher H., Hendrickse P.W., Pereira M.G., Bowen T.S. (2023). Skeletal muscle atrophy, regeneration, and dysfunction in heart failure: impact of exercise training. J Sport Health Sci.

[32] Theocharis A.D., Skandalis S.S., Gialeli C., Karamanos N.K. (2016). Extracellular matrix structure. Adv Drug Deliv Rev.

[33] Wohlgemuth R.P., Brashear S.E., Smith L.R. (2023). Alignment, cross linking, and beyond: a collagen architect's guide to the skeletal muscle extracellular matrix. Am J Physiol Cell Physiol.

[34] Kechagia J.Z., Ivaska J., Roca-Cusachs P. (2019). Integrins as biomechanical sensors of the microenvironment. Nat Rev Mol Cell Biol.

[35] Soslow J.H., Xu M., Slaughter J.C., Crum K., Chew J.D., Burnette W.B. (2019). The role of matrix metalloproteinases and tissue inhibitors of metalloproteinases in duchenne muscular dystrophy cardiomyopathy. J Card Fail.

[36] Kovanen V. (2002). Intramuscular extracellular matrix: complex environment of muscle cells. Exerc Sport Sci Rev.

[37] Cescon M., Gattazzo F., Chen P., Bonaldo P. (2015). Collagen VI at a glance. J Cell Sci.

[38] Gillies A.R., Lieber R.L. (2011). Structure and function of the skeletal muscle extracellular matrix. Muscle Nerve.

[39] Kragstrup T.W., Kjaer M., Mackey A.L. (2011). Structural, biochemical, cellular, and functional changes in skeletal muscle extracellular matrix with aging. Scand J Med Sci Sports.

[40] Shavlakadze T., Xiong K., Mishra S., McEwen C., Gadi A., Wakai M. (2023). Age-related gene expression signatures from limb skeletal muscles and the diaphragm in mice and rats reveal common and species-specific changes. Skeletal Muscle.

[41] Kim H., Kim M.C., Asada H.H. (2019). Extracellular matrix remodelling induced by alternating electrical and mechanical stimulations increases the contraction of engineered skeletal muscle tissues. Sci Rep.

[42] Brightwell C.R., Latham C.M., Thomas N.T., Keeble A.R., Murach K.A., Fry C.S. (2022). A glitch in the matrix: the pivotal role for extracellular matrix remodeling during muscle hypertrophy. Am J Physiol Cell Physiol.

[43] Fede C., Fan C., Pirri C., Petrelli L., Biz C., Porzionato A. (2022). The effects of aging on the intramuscular connective tissue. Int J Mol Sci.

[44] Lieber R.L., Fridén J. (2019). Muscle contracture and passive mechanics in cerebral palsy. J Appl Physiol.

[45] Velleman S.G. (2012). Meat Science and Muscle Biology Symposium: extracellular matrix regulation of skeletal muscle formation. J Anim Sci.

[46] Droguett R., Cabello-Verrugio C., Riquelme C., Brandan E. (2006). Extracellular proteoglycans modify TGF-beta bio-availability attenuating its signaling during skeletal muscle differentiation. Matrix Biol.

[47] Ahmad S., Jan A.T., Baig M.H., Lee E.J., Choi I. (2017). Matrix gla protein: an extracellular matrix protein regulates myostatin expression in the muscle developmental program. Life Sci.

[48] Hjorth M., Norheim F., Meen A.J., Pourteymour S., Lee S., Holen T. (2015). The effect of acute and long-term physical activity on extracellular matrix and serglycin in human skeletal muscle. Phys Rep.

[49] Lehti T.M., Silvennoinen M., Kivelä R., Kainulainen H., Komulainen J. (2006). Effects of streptozotocin-induced diabetes and physical training on gene expression of extracellular matrix proteins in mouse skeletal muscle. Am J Physiol Endocrinol Metab.

[50] Elosegui-Artola A. (2021). The extracellular matrix viscoelasticity as a regulator of cell and tissue dynamics. Curr Opin Cell Biol.

[51] Shi N., Wang J., Tang S., Zhang H., Wei Z., Li A. (2024). Matrix nonlinear viscoelasticity regulates skeletal myogenesis through MRTF nuclear localization and nuclear mechanotransduction. Small.

[52] Winograd-Katz S.E., Fässler R., Geiger B., Legate K.R. (2014). The integrin adhesome: from genes and proteins to human disease. Nat Rev Mol Cell Biol.

[53] Liu J., Burkin D.J., Kaufman S.J. (2008). Increasing alpha 7 beta 1-integrin promotes muscle cell proliferation, adhesion, and resistance to apoptosis without changing gene expression. Am J Physiol Cell Physiol.

[54] Boppart M.D., Burkin D.J., Kaufman S.J. (2006). Alpha7beta1-integrin regulates mechanotransduction and prevents skeletal muscle injury. Am J Physiol Cell Physiol.

[55] Boppart M.D., Mahmassani Z.S. (2019). Integrin signaling: linking mechanical stimulation to skeletal muscle hypertrophy. Am J Physiol Cell Physiol.

[56] Lueders T.N., Zou K., Huntsman H.D., Meador B., Mahmassani Z., Abel M. (2011). The α7β1-integrin accelerates fiber hypertrophy and myogenesis following a single bout of eccentric exercise. Am J Physiol Cell Physiol.

[57] Calve S., Isaac J., Gumucio J.P., Mendias C.L. (2012). Hyaluronic acid, HAS1, and HAS2 are significantly upregulated during muscle hypertrophy [published correction appears in Am J Physiol Cell Physiol. 2012 Oct 15;303(8):C895-6]. Am J Physiol Cell Physiol.

[58] Leng Y., Abdullah A., Wendt M.K., Calve S. (2019). Hyaluronic acid, CD44 and RHAMM regulate myoblast behavior during embryogenesis. Matrix Biol.

[59] Amir A., Kim S., Stecco A., Jankowski M.P., Raghavan P. (2022). Hyaluronan homeostasis and its role in pain and muscle stiffness. Pharm Manag PM R.

[60] Turner N.J., Yates A.J., Weber D.J., Qureshi I.R., Stolz D.B., Gilbert T.W. (2010). Xenogeneic extracellular matrix as an inductive scaffold for regeneration of a functioning musculotendinous junction. Tissue Eng.

[61] Valentin J.E., Turner N.J., Gilbert T.W., Badylak S.F. (2010). Functional skeletal muscle formation with a biologic scaffold. Biomaterials.

[62] Agrawal V., Brown B.N., Beattie A.J., Gilbert T.W., Badylak S.F. (2009). Evidence of innervation following extracellular matrix scaffold-mediated remodelling of muscular tissues. J Tissue Eng Regen Med.

[63] Raffa P., Scattolini V., Gerli M.F.M., Perin S., Cui M., De Coppi P. (2020). Decellularized skeletal muscles display neurotrophic effects in three-dimensional organotypic cultures. Stem Cells Transl Med.

[64] Trevisan C., Maghin E., Dedja A., Caccin P., de Cesare N., Franzin C. (2019). Allogenic tissue-specific decellularized scaffolds promote long-term muscle innervation and functional recovery in a surgical diaphragmatic hernia model. Acta Biomater.

[65] Urciuolo A., Urbani L., Perin S., Maghsoudlou P., Scottoni F., Gjinovci A. (2018). Decellularised skeletal muscles allow functional muscle regeneration by promoting host cell migration. Sci Rep.

[66] Hoshiba T., Yokoyama N. (2020). Decellularized extracellular matrices derived from cultured cells at stepwise myogenic stages for the regulation of myotube formation. Biochim Biophys Acta Mol Cell Res.

[67] Wu Young MY., Dolivo D.M., Hong S.J., Iyer H., Mustoe T.A., Galiano R.D. (2020). Decellularized fetal matrix suppresses fibrotic gene expression and promotes myogenesis in a rat model of volumetric muscle loss. Plast Reconstr Surg.

[68] Barajaa M.A., Otsuka T., Ghosh D., Kan H.M., Laurencin C.T. (2024). Development of porcine skeletal muscle extracellular matrix-derived hydrogels with improved properties and low immunogenicity. Proc Natl Acad Sci U S A.

[69] Tang S.W., Yuen W., Kaur I., Pang S.W., Voelcker N.H., Lam Y.W. (2020). Capturing instructive cues of tissue microenvironment by silica bioreplication. Acta Biomater.

[70] Goldman S.M., Corona B.T. (2017). Co-delivery of micronized urinary bladder matrix damps regenerative capacity of minced muscle grafts in the treatment of volumetric muscle loss injuries. PLoS One.

[71] Zhu M., Li W., Dong X., Yuan X., Midgley A.C., Chang H. (2019). In vivo engineered extracellular matrix scaffolds with instructive niches for oriented tissue regeneration. Nat Commun.

[72] Jiao A., Moerk C.T., Penland N., Perla M., Kim J., Smith A.S.T. (2018). Regulation of skeletal myotube formation and alignment by nanotopographically controlled cell-secreted extracellular matrix. J Biomed Mater Res.

[73] Smoak M.M., Hogan K.J., Grande-Allen K.J., Mikos A.G. (2021). Bioinspired electrospun dECM scaffolds guide cell growth and control the formation of myotubes. Sci Adv.

[74] Kasukonis B., Kim J., Brown L., Jones J., Ahmadi S., Washington T. (2016). Codelivery of infusion decellularized skeletal muscle with minced muscle autografts improved recovery from volumetric muscle loss injury in a rat model. Tissue Eng.

[75] Reed C., Huynh T., Schluns J., Phelps P., Hestekin J., Wolchok J.C. (2024). Cell-derived extracellular matrix fiber scaffolds improve recovery from volumetric muscle loss. Tissue Eng.

[76] Conconi M.T., De Coppi P., Bellini S., Zara G., Sabatti M., Marzaro M. (2005). Homologous muscle acellular matrix seeded with autologous myoblasts as a tissue-engineering approach to abdominal wall-defect repair. Biomaterials.

[77] Olson L.C., Redden J.T., Gilliam L., Nguyen T.M., Vossen J.A., Cohen D.J. (2022). Human adipose-derived stromal cells delivered on decellularized muscle improve muscle regeneration and regulate RAGE and P38 MAPK. Bioengineering.

[78] Qiu X., Liu S., Zhang H., Zhu B., Su Y., Zheng C. (2018). Mesenchymal stem cells and extracellular matrix scaffold promote muscle regeneration by synergistically regulating macrophage polarization toward the M2 phenotype. Stem Cell Res Ther.

[79] Corona B.T., Ward C.L., Baker H.B., Walters T.J., Christ G.J. (2014). Implantation of in vitro tissue engineered muscle repair constructs and bladder acellular matrices partially restore in vivo skeletal muscle function in a rat model of volumetric muscle loss injury. Tissue Eng.

[80] Ay B., Karaoz E., Kesemenli C.C., Kenar H. (2017). Skeletal muscle patch engineering on synthetic and acellular human skeletal muscle originated scaffolds. J Biomed Mater Res.

[81] Rajabi S., Jalili-Firoozinezhad S., Ashtiani M.K., Le Carrou G., Tajbakhsh S., Baharvand H. (2018). Effect of chemical immobilization of SDF-1α into muscle-derived scaffolds on angiogenesis and muscle progenitor recruitment. J Tissue Eng Regen Med.

[82] Lee H., Ju Y.M., Kim I., Elsangeedy E., Lee J.H., Yoo J.J. (2020). A novel decellularized skeletal muscle-derived ECM scaffolding system for in situ muscle regeneration. Methods.

[83] Kim W., Lee H., Lee J., Atala A., Yoo J.J., Lee S.J. (2020). Efficient myotube formation in 3D bioprinted tissue construct by biochemical and topographical cues. Biomaterials.

[84] Cvetkovic C., Rich M.H., Raman R., Kong H., Bashir R. (2017). A 3D-printed platform for modular neuromuscular motor units. Microsyst Nanoeng.

[85] Lee H., Kim W., Lee J., Yoo J.J., Kim G.H., Lee S.J. (2019). Effect of hierarchical scaffold consisting of aligned dECM nanofibers and poly(lactide-co-glycolide) struts on the orientation and maturation of human muscle progenitor cells. ACS Appl Mater Interfaces.

[86] Patel K.H., Dunn A.J., Talovic M., Haas G.J., Marcinczyk M., Elmashhady H. (2019). Aligned nanofibers of decellularized muscle ECM support myogenic activity in primary satellite cells in vitro. Biomed Mater.

[87] Ker E.D., Nain A.S., Weiss L.E., Wang J., Suhan J., Amon C.H. (2011). Bioprinting of growth factors onto aligned sub-micron fibrous scaffolds for simultaneous control of cell differentiation and alignment. Biomaterials.

[88] Yi H., Forsythe S., He Y., Liu Q., Xiong G., Wei S. (2017). Tissue-specific extracellular matrix promotes myogenic differentiation of human muscle progenitor cells on gelatin and heparin conjugated alginate hydrogels. Acta Biomater.

[89] Zahari N.K., Idrus R.B.H., Chowdhury S.R. (2017). Laminin-coated poly(methyl methacrylate) (PMMA) nanofiber scaffold facilitates the enrichment of skeletal muscle myoblast population. Int J Mol Sci.

[90] Besser R.R., Bowles A.C., Alassaf A., Carbonero D., Claure I., Jones E. (2020). Enzymatically crosslinked gelatin-laminin hydrogels for applications in neuromuscular tissue engineering. Biomater Sci.

[91] West C., Tobo C., Au J., Johnson D., Mottaleb M.A., Robinson J. (2023). Combined application of biosponges and an antifibrotic agent for the treatment of volumetric muscle loss. Am J Physiol Cell Physiol.

[92] Silva Garcia J.M., Panitch A., Calve S. (2019). Functionalization of hyaluronic acid hydrogels with ECM-derived peptides to control myoblast behavior. Acta Biomater.

[93] Boonen K.J., van der Schaft D.W., Baaijens F.P., Post M.J. (2011). Interaction between electrical stimulation, protein coating and matrix elasticity: a complex effect on muscle fibre maturation. J Tissue Eng Regen Med.

[94] Yang H.S., Lee B., Tsui J.H., Macadangdang J., Jang S.Y., Im S.G. (2016). Electroconductive nanopatterned substrates for enhanced myogenic differentiation and maturation. Adv Healthcare Mater.

[95] Maimaiti D., Ge X., Wang C., Liu J., Yang G., Zhang D. (2024). Extracellular matrix-mimicking cryogels composed of methacrylated fucoidan enhance vascularized skeletal muscle regeneration following volumetric muscle loss. Int J Biol Macromol.

[96] Arab W., Rauf S., Al-Harbi O., Hauser C.A.E. (2018). Novel ultrashort self-assembling peptide bioinks for 3D culture of muscle myoblast cells. Int J Bioprint.

[97] Carnes M.E., Pins G.D. (2020). Etching anisotropic surface topography onto fibrin microthread scaffolds for guiding myoblast alignment. J Biomed Mater Res B Appl Biomater.

[98] Chen S., Nakamoto T., Kawazoe N., Chen G. (2015). Engineering multi-layered skeletal muscle tissue by using 3D microgrooved collagen scaffolds. Biomaterials.

[99] Jana S., Leung M., Chang J., Zhang M. (2014). Effect of nano- and micro-scale topological features on alignment of muscle cells and commitment of myogenic differentiation. Biofabrication.

[100] Chen H., Zhong J., Wang J., Huang R., Qiao X., Wang H. (2019). Enhanced growth and differentiation of myoblast cells grown on E-jet 3D printed platforms. Int J Nanomed.

[101] Kratzer S., Arkudas A., Himmler M., Schubert D.W., Schneidereit D., Bauer J. (2022). Vascularization of poly-ε-caprolactone-collagen I-nanofibers with or without sacrificial fibers in the neurotized arteriovenous loop model. Cells.

[102] Pacilio S., Costa R., Papa V., Rodia M.T., Gotti C., Pagnotta G. (2023). Electrospun poly(L-lactide-co-ε-caprolactone) scaffold potentiates C2C12 myoblast bioactivity and acts as a stimulus for cell commitment in skeletal muscle myogenesis. Bioengineering.

[103] Tonda-Turo C., Ruini F., Ramella M., Boccafoschi F., Gentile P., Gioffredi E. (2017). Non-covalently crosslinked chitosan nanofibrous mats prepared by electrospinning as substrates for soft tissue regeneration. Carbohydr Polym.

[104] Aparicio-Collado J.L., Molina-Mateo J., Cabanilles C.T., Vidaurre A., Salesa B., Serrano-Aroca Á. (2022). Pro-myogenic environment promoted by the synergistic effect of conductive polymer nanocomposites combined with extracellular zinc ions. Biology.

[105] Patel A., Xue Y., Hartley R., Sant V., Eles J.R., Cui X.T. (2018). Hierarchically aligned fibrous hydrogel films through microfluidic self-assembly of graphene and polysaccharides. Biotechnol Bioeng.

[106] Zhang Y., Le Friec A., Chen M. (2021). 3D anisotropic conductive fibers electrically stimulated myogenesis. Int J Pharm.

[107] Srisuk P., Berti F.V., da Silva L.P., Marques A.P., Reis R.L., Correlo V.M. (2018). Electroactive gellan gum/polyaniline spongy-like hydrogels. ACS Biomater Sci Eng.

[108] Zhang Y., Zhang Z., Wang Y., Su Y., Chen M. (2020). 3D myotube guidance on hierarchically organized anisotropic and conductive fibers for skeletal muscle tissue engineering. Mater Sci Eng C.

[109] Schilling B.K., Baker J.S., Komatsu C., Marra K.G. (2021). Intramuscular injection of skeletal muscle derived extracellular matrix mitigates denervation atrophy after sciatic nerve transection. J Tissue Eng.

[110] Estrellas K.M., Chung L., Cheu L.A., Sadtler K., Majumdar S., Mula J. (2018). Biological scaffold-mediated delivery of myostatin inhibitor promotes a regenerative immune response in an animal model of Duchenne muscular dystrophy. J Biol Chem.

[111] Rooney J.E., Gurpur P.B., Burkin D.J. (2009). Laminin-111 protein therapy prevents muscle disease in the mdx mouse model for Duchenne muscular dystrophy. Proc Natl Acad Sci U S A.

[112] Garg K., Mahmassani Z.S., Dvoretskiy S., Valero M.C., Huntsman H.D., Lapp S. (2021). Laminin-111 improves the anabolic response to mechanical load in aged skeletal muscle. J Gerontol A Biol Sci Med Sci.

[113] Fallon J.R., McNally E.M. (2018). Non-glycanated biglycan and LTBP4: leveraging the extracellular matrix for duchenne muscular dystrophy therapeutics. Matrix Biol.

[114] Fix D.K., Mahmassani Z.S., Petrocelli J.J., de Hart N.M.M.P., Ferrara P.J., Painter J.S. (2021). Reversal of deficits in aged skeletal muscle during disuse and recovery in response to treatment with a secrotome product derived from partially differentiated human pluripotent stem cells. Geroscience.

[115] Alexeev V., Arita M., Donahue A., Bonaldo P., Chu M., Igoucheva O. (2014). Human adipose-derived stem cell transplantation as a potential therapy for collagen VI-related congenital muscular dystrophy. Stem Cell Res Ther.

[116] Marrosu E., Ala P., Muntoni F., Zhou H. (2017). Gapmer antisense oligonucleotides suppress the mutant allele of COL6A3 and restore functional protein in ullrich muscular dystrophy. Mol Ther Nucleic Acids.

[117] Meinen S., Lin S., Thurnherr R., Erb M., Meier T., Rüegg M.A. (2011). Apoptosis inhibitors and mini-agrin have additive benefits in congenital muscular dystrophy mice. EMBO Mol Med.

[118] Qiao C., Li J., Zhu T., Draviam R., Watkins S., Ye X. (2005). Amelioration of laminin-alpha2-deficient congenital muscular dystrophy by somatic gene transfer of miniagrin. Proc Natl Acad Sci U S A.

[119] Domi T., Porrello E., Velardo D., Capotondo A., Biffi A., Tonlorenzi R. (2015). Mesoangioblast delivery of miniagrin ameliorates murine model of merosin-deficient congenital muscular dystrophy type 1A. Skeletal Muscle.

[120] McKee K.K., Yurchenco P.D. (2022). Amelioration of muscle and nerve pathology of Lama2-related dystrophy by AAV9-laminin-αLN linker protein. JCI Insight.

[121] Packer D., Martin P.T. (2021). Micro-laminin gene therapy can function as an inhibitor of muscle disease in the dy^W^ mouse model of MDC1A. Mol Ther Methods Clin Dev.

[122] Swiderski K., Brock C.J., Trieu J., Chee A., Thakur S.S., Baum D.M. (2021). Phosphorylation of ERK and dystrophin S3059 protects against inflammation-associated C2C12 myotube atrophy. Am J Physiol Cell Physiol.

[123] Marshall J.L., Kwok Y., McMorran B.J., Baum L.G., Crosbie-Watson R.H. (2013). The potential of sarcospan in adhesion complex replacement therapeutics for the treatment of muscular dystrophy. FEBS J.

[124] Vidal B., Ardite E., Suelves M., Ruiz-Bonilla V., Janué A., Flick M.J. (2012). Amelioration of Duchenne muscular dystrophy in mdx mice by elimination of matrix-associated fibrin-driven inflammation coupled to the αMβ2 leukocyte integrin receptor. Hum Mol Genet.

[125] McRae N., Forgan L., McNeill B., Addinsall A., McCulloch D., Van der Poel C. (2017). Glucocorticoids improve myogenic differentiation in vitro by suppressing the synthesis of versican, a transitional matrix protein overexpressed in dystrophic skeletal muscles. Int J Mol Sci.

[126] Addinsall A.B., Forgan L.G., McRae N.L., Kelly R.W., McDonald P.L., McNeill B. (2020). Treatment of dystrophic mdx mice with an ADAMTS-5 specific monoclonal antibody increases the ex vivo strength of isolated fast twitch hindlimb muscles. Biomolecules.

[127] Wang Y., Xiao Y., Zheng Y., Yang L., Wang D. (2021). An anti-ADAMTS1 treatment relieved muscle dysfunction and fibrosis in dystrophic mice. Life Sci.

[128] Ceco E., McNally E.M. (2013). Modifying muscular dystrophy through transforming growth factor-β. FEBS J.

[129] Demonbreun A.R., Fallon K.S., Oosterbaan C.C., Vaught L.A., Reiser N.L., Bogdanovic E. (2021). Anti-latent TGFβ binding protein 4 antibody improves muscle function and reduces muscle fibrosis in muscular dystrophy. Sci Transl Med.

[130] Ito M., Ehara Y., Li J., Inada K., Ohno K. (2017). Protein-anchoring therapy of biglycan for mdx mouse model of duchenne muscular dystrophy. Hum Gene Ther.

[131] Ogura Y., Tajrishi M.M., Sato S., Hindi S.M., Kumar A. (2014). Therapeutic potential of matrix metalloproteinases in Duchenne muscular dystrophy. Front Cell Dev Biol.

[132] Hindi S.M., Shin J., Ogura Y., Li H., Kumar A. (2013). Matrix metalloproteinase-9 inhibition improves proliferation and engraftment of myogenic cells in dystrophic muscle of mdx mice. PLoS One.

[133] Bobadilla Muñoz M., Orbe J., Abizanda G., Machado F.J.D., Vilas A., Ullate-Agote A. (2023). Loss of the matrix metalloproteinase-10 causes premature features of aging in satellite cells. Front Cell Dev Biol.

[134] Bobadilla M., Sáinz N., Rodriguez J.A., Abizanda G., Orbe J., de Martino A. (2014). MMP-10 is required for efficient muscle regeneration in mouse models of injury and muscular dystrophy. Stem Cell.

[135] Choi A., Park S.E., Jeong J.B., Choi S.J., Oh S.Y., Ryu G.H. (2020). Anti-fibrotic effect of human Wharton's jelly-derived mesenchymal stem cells on skeletal muscle cells, mediated by secretion of MMP-1. Int J Mol Sci.

[136] Burkin D.J., Wallace G.Q., Nicol K.J., Kaufman D.J., Kaufman S.J. (2001). Enhanced expression of the alpha 7 beta 1 integrin reduces muscular dystrophy and restores viability in dystrophic mice. J Cell Biol.

[137] Heller K.N., Montgomery C.L., Janssen P.M., Clark K.R., Mendell J.R., Rodino-Klapac L.R. (2013). AAV-mediated overexpression of human α7 integrin leads to histological and functional improvement in dystrophic mice. Mol Ther.

[138] Liu J., Milner D.J., Boppart M.D., Ross R.S., Kaufman S.J. (2012). β1D chain increases α7β1 integrin and laminin and protects against sarcolemmal damage in mdx mice. Hum Mol Genet.

[139] Sarathy A., Wuebbles R.D., Fontelonga T.M., Tarchione A.R., Mathews Griner L.A., Heredia D.J. (2017). SU9516 increases α7β1 integrin and ameliorates disease progression in the mdx mouse model of duchenne muscular dystrophy. Mol Ther.

[140] Williams A.S., Kang L., Wasserman D.H. (2015). The extracellular matrix and insulin resistance. Trends Endocrinol Metabol.

